# Screening for Triterpenoid Saponins in Plants Using Hyphenated Analytical Platforms

**DOI:** 10.3390/molecules21121614

**Published:** 2016-11-24

**Authors:** Bekzod Khakimov, Li Hong Tseng, Markus Godejohann, Søren Bak, Søren Balling Engelsen

**Affiliations:** 1 Department of Food Science, Faculty of Science, University of Copenhagen, Rolighedsvej 16, DK-1958 Frederiksberg C, Denmark; se@food.ku.dk; 2 Department of Plant and Environmental Sciences, Faculty of Science, University of Copenhagen, Rolighedsvej 16, DK-1958 Frederiksberg C, Denmark; bak@plen.ku.dk; 3 Bruker BioSpin GmbH, 76287 Rheinstetten, Germany; lihong.tseng@bruker.com (L.H.T.); Markus.Godejohann@bruker.com (M.G.)

**Keywords:** time slice LC-SPE-NMR/MS, GC-MS, LC-MS/MS, triterpenoid saponins, *Barbarea vulgaris*

## Abstract

Recently the number of studies investigating triterpenoid saponins has drastically increased due to their diverse and potentially attractive biological activities. Currently the literature contains chemical structures of few hundreds of triterpenoid saponins of plant and animal origin. Triterpenoid saponins consist of a triterpene aglycone with one or more sugar moieties attached to it. However, due to similar physico-chemical properties, isolation and identification of a large diversity of triterpenoid saponins remain challenging. This study demonstrates a methodology to screen saponins using hyphenated analytical platforms, GC-MS, LC-MS/MS, and LC-SPE-NMR/MS, in the example of two different phenotypes of the model plant *Barbarea vulgaris* (winter cress), glabrous (G) and pubescent (P) type that are known to differ by their insect resistance. The proposed methodology allows for detailed comparison of saponin profiles from intact plant extracts as well as saponin aglycone profiles from hydrolysed samples. Continuously measured 1D proton NMR data during LC separation along with mass spectrometry data revealed significant differences, including contents of saponins, types of aglycones and numbers of sugar moieties attached to the aglycone. A total of 49 peaks were tentatively identified as saponins from both plants; they are derived from eight types of aglycones and with 2–5 sugar moieties. Identification of two previously known insect-deterrent saponins, hederagenin cellobioside and oleanolic acid cellobioside, demonstrated the applicability of the methodology for relatively rapid screening of bioactive compounds.

## 1. Introduction

Triterpenoid saponins consist of a triterpene aglycone, with 30 carbon atoms, with one or more sugar moieties (including hexoses, methylpentoses, and pentoses) attached to the aglycone [1]. Triterpenoid saponins are secondary metabolites synthesized in plant and mammalian cells. Several studies have reported on the role of triperpenoid saponins as natural defence compounds in plants [2,3,4,5], and some members of triterpenoid saponins have also been found to possess beneficial pharmacological properties [6,7,8]. The literature contains structures of several hundred saponins isolated from various sources including plants and animals. Saponins exhibit diverse biological activities, and a number of studies investigating biosynthetic pathways and roles of this class of metabolites in the growth, development and physiology of host organisms has increased [9,10,11]. Recently triterpenoid saponins also became central target compounds for developing natural pesticides and insecticides for crop management in agriculture. Saponins are amphiphilic in nature, due to the hydrophilic sugar moieties and the lipophilic triterpenoid aglycone. For this reason, the extraction and qualitative and quantitative analysis of saponins might be challenging from complex sample matrices such as plants and must be optimized prior to qualitative and quantitative analysis. This often includes a selection of an appropriate solvent composition depending on the physico-chemical properties of the investigated saponins.

Despite the fact that LC-MS is the method of choice for the analysis of saponins, often the chromatographic resolution power is not sufficient to separate structurally similar saponins. The LC-MS analysis of saponins may become even more challenging when samples contain a wide range of hyper-glycosylated saponins (e.g., saponins with three and more sugar moieties) with a similar molecular weight (MW) and/or glycosylation patterns. Nevertheless, LC-MS provides the richest information about the chemical structures of saponins obtained directly from complex mixtures. Structural characterization of saponins can be further improved when tandem mass spectrometry, for example LC-MS/MS, is applied [12]. This will provide unique fragmentation patterns of saponins including the number and type of sugar moieties attached to the aglycone. While GC-MS analysis of saponins is impossible due to high MW and boiling points of the molecules, the triterpenoid aglycones are commonly analysed using the GC-MS. The lipophilic nature and thermal stability of triterpenoid aglycones makes them suitable for GC-MS analysis providing high detection sensitivity and a characteristic electron impact-mass spectrum (EI-MS). In order to screen for triterpenoid aglycone molecules, samples are often hydrolysed to cleave off sugar moieties attached to the aglycone. This procedure is usually performed either by chemical, using acidic or alkaline, or enzymatic hydrolysis. Such protocols are known to be complex, time consuming and require careful optimization in order to improve method reproducibility. Following hydrolysis, the free and liberated aglycone containing samples are derivatized using trimethylsilylation before injection into a GC-MS [13]. Nuclear magnetic resonance (NMR) spectroscopy is another complementary analytical platform to GC-MS and LC-MS for the analysis of saponins. Despite the fact that the sensitivity of NMR is inferior to GC-MS and LC-MS, it can, with practically no sample preparation, quantitatively detect all metabolites, irrespective of their volatility, polarity, size, and chemical structure, provided that they possess chemical elements with non-zero spin quantum number, such as ^1^H, ^13^C, ^31^P, and ^15^N. Since the proton is the most suitable nucleus (most sensitive, producing sharp and informative NMR signals and allowing rapid data acquisition) for NMR and it is a part of the majority of metabolites, NMR based metabolomics is most often performed using proton nuclei. The main advantages of NMR over mass spectrometry based methods are that it is non-destructive, fast, reproducible and requires much less labour for sample preparation. However, due to the relatively low concentration of saponins when compared to other metabolites present in complex plant tissue matrices, the detection of saponins using NMR can be challenging without prior sample cleaning and saponin enrichment.

This study demonstrates the application of hyphenated analytical platforms, including GC-MS, LC-MS/MS, NMR and time slice LC-SPE-NMR/MS, for the qualitative and relative quantitative analysis of triterpenoid saponins in plant leaf extracts of *B. vulgaris*. Two different genotypes of *B. vulgaris* plants, glabrous (G-type) and pubescent (P-type), that differ by their saponin content and insect resistance toward *Phyllotreta nemorum*, large striped flea beetle, were screened using a global metabolite extract and a saponin enriched extract. The study shows that the complete structure elucidation of saponins directly from complex samples mixtures is challenging even when multiple hyphenated platforms are used. However, a significant amount of structural information, MW of aglycones and number and types of sugar moieties can be gained by combining the information obtained from the different platforms. 

## 2. Results and Discussion

### 2.1. Untargeted Metabolomics for Analysis of Triterpenoid Saponins in B. vulgaris

**Figure 1:**
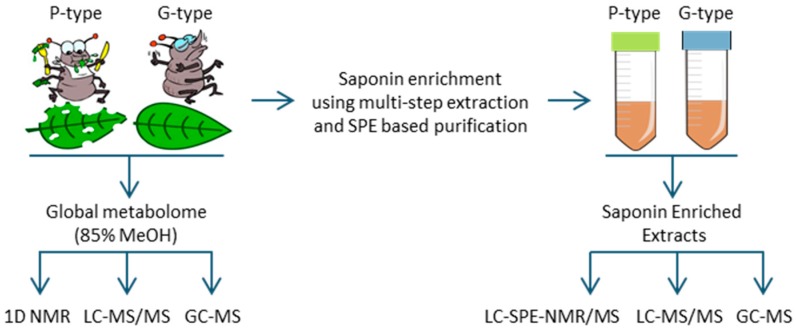
An overview of the study design. A global metabolite extract based on 85% methanol was employed to screen for triterpenoid saponins using three different analytical platforms, GC-MS, LC-MS/MS and NMR. Saponin enriched extracts based on SPE based purification of the 80% methanol extract were screened using LC-SPE-NMR/MS, LC-MS/MS, and GC-MS.

In this study, it was attempted to develop a single metabolomics protocol covering as broad range of metabolites as possible, including saponins, from a leaf tissue of *B. vulgaris*. A single metabolite extraction method based on 85% methanol was developed and employed for three different analytical platforms, including GC-MS, LC-MS and NMR (Figure 1) [14]. A previous LC-MS metabolomics study performed on the same methanol extracts of 160 F2 plants derived from a crossing of parental G- and P-type *B. vulgaris* plants resulted in the identification of saponins that were related to the plants’ resistance against the *P. nemorum* larvae [2]. The follow up study further scrutinized the same LC-MS data using a different approach based on PARAllel FACtor Analysis 2 (PARAFAC2) [15,16], which allowed the deconvolution of even very elusive LC-MS peaks directly from the raw data. This approach enabled the tentative identification of saponins responsible for discrimination of F2 plants based on their insect resistance and further insights into interactions between the plants’ metabolome and insect resistance [17]. Among these metabolites, hederagenin cellobioside, oleanolic acid cellobioside, 4-epihederagenin cellobioside, gypsogenin cellobioside, and a few other unknown saponins were found to be the most deterrent towards the larvae of *P. nemorum*. This analysis was based on PLS regression relating the metabolomics and insect resistance data. 

Despite the fact that untargeted LC-MS analysis provides rich metabolomics data, the sensitivity and selectivity of a given method vary for different chemical classes. Quantification of metabolites may often be a challenge due to ion suppression and/or complex sample matrix effects. The physico-chemical properties of triterpenoid saponins, including their chemical structure, molecular size and polarity largely influence the ionization efficiency of molecules during the electrospray ionization (ESI) in LC-MS, which in turn determines the sensitivity of the analysis. Thus, the use of an untargeted LC-MS metabolomics protocol may not be an optimal approach for quantification of triterpenoid saponins from complex plant extracts without prior method optimization. The LC-MS method applied in this study was developed to emphasise detection of triterpenoid saponins from the 85% methanol extract of *B. vulgaris* plant leaves. Tandem mass spectrometry was used for the characterization of the type and number of sugar moieties as well as the MW of the triterpenoid aglycone [14]. This approach allowed tentative characterization of a total of 73 saponin peaks from G- and P-type *B. vulgaris*. Most of these saponins had an aglycone MW of 448, 456, 458, 472, or 474 Da and included two to five sugar moieties, primarily hexose (162 Da), methylpentose (146 Da), and pentose (132 Da) [14].

In order to screen for triterpenoid aglycones using GC-MS, *B. vulgaris* plant extracts were hydrolysed using hydrochloric acid and the liberated aglycones subsequently trimethylsilylated using a new derivatization method developed based on trimethylsilyl cyanide (TMSCN) [13]. Prior to the GC-MS analysis of *B. vulgaris* triterpenoid aglycones, the derivatization method was tested on the dilution series of several known triterpenoid aglycone standards including hederagenin, oleanolic acid and betulinic acid, which showed a correlation coefficient (r^2^) of 0.95 or higher [14]. The unique EI-MS patterns and retention indices (RIs) of triterpenoid aglycones obtained from the GC-MS analysis allow for unambiguous identification. A design of an experiment (DoE) approach was then used to optimize the protocol including extraction (solvent composition, time and temperature), hydrolyzation (time, temperature) and trimethylsilylation (time, temperature) parameters. A 2^7-3^ fractional factorial experiment was designed and all seven parameters of the protocol were varied at three levels resulting in a total of 19 experiments covering as broad experimental domain as possible [14]. The S/N ratio and detection reproducibility of the aglycones, hederagenin and oleanolic acid were evaluated for all experiments performed within the DoE and the parameters of the optimal protocol were found as follows: extraction (85% methanol, 5 min and 100 °C), acidic hydrolyzation (1 h and 99 °C), and trimethylsilylation parameters (40 min and 40 °C). More detailed results on aglycones profiles of *B. vulgaris* are discussed in Section 2.3.

**Figure 2:**
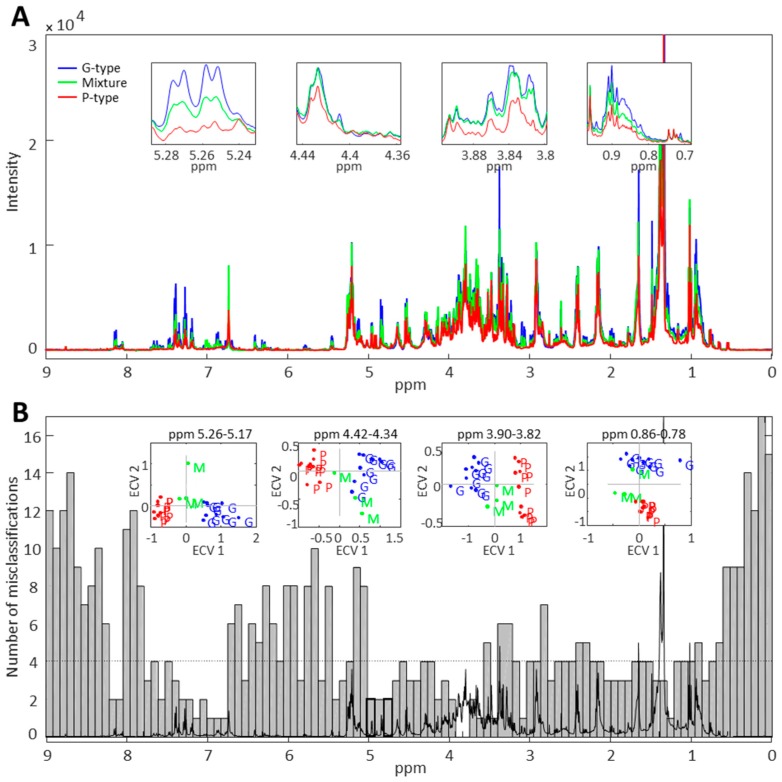
1D proton NMR analysis of global metabolome of leaves of G- and P-type *B. vulgaris* using 85% methanol extracts. (**A**) Mean of 1D proton NMR spectra of 10 G-type, 10 P-type, and 3 mixture (50% G- and 50% P-type plants) samples; (**B**) Interval based extended canonical variate analysis (iECVA) of the raw NMR data containing 23 spectra. The iECVA analysis depicted spectral regions that were most discriminative between G- and P-type plants. iECVA was performed on 100 equal size intervals across the entire NMR spectra.

1D proton NMR spectra recorded on 85% methanol extracts of G- and P-type *B. vulgaris* plants as well as on their combined samples (50% G- and 50% P-type) revealed spectral regions that clearly differentiate the two genotypes (Figure 2A). The NMR spectra measured on complex plant extracts represent signals from the most abundant metabolites while signals of low level metabolites remain undetected or largely hidden by dominating metabolites. In addition, due to the complexity of the sample matrix, the NMR signals derived from different molecules overlap and may hamper data interpretation. Thus, it is almost impossible to identify well resolved NMR signals of saponins from such a complex sample mixture. However, significant differences in the NMR spectra of G- and P-type plants were pronounced in the spectral regions that correspond to chemical shifts of characteristic signals of saponins. These include the aliphatic region (0.7–1.2 ppm) with singlets derived from the methyl groups of triterpenoid aglycones and the anomeric region (4.3–4.5 ppm) with doublets corresponding to anomeric protons of sugar moieties (Figure S1). Despite being crowded and difficult to interpret, the spectral region corresponding to 3.6–3.9 ppm, which represent the protons of sugar moieties in saponins, also show a clear difference between the two genotypes. Employment of the interval based extended canonical variate analysis (iECVA) [18] on the raw NMR dataset enabled the identification of spectral regions that can discriminate between the two plant genotypes. The resulting discriminative spectral regions mostly represent saponin peaks (Figure 2B). In addition to aliphatic and anomeric regions, the spectral region from 5.2–5.3 ppm was found to be the most powerful region for discrimination. In NMR spectra of saponins derived from β-amyrin, α-amyrin and other similar triterpenoid aglycones, the spectral region of 5.2–5.3 ppm corresponds to the triplet from the methine group at C12 position (Figure S1). This suggests that one of the main differences between G- and P-type plant saponins might be a special type of aglycone and the data presented in this study indicates that saponins of the G-type plant contain primarily β-amyrin, α-amyrin, and/or other similar aglycones. This finding is in agreement with previous studies that showed isolation and identification of several saponins from the G-type *B. vulgaris*, where all saponins were based on β-amyrin such as hederagenin cellobioside and oleanolic acid cellobioside [3,4,19].

### 2.2. Time Slice LC-SPE-NMR/MS Experiment on Saponin Enriched Extracts from G- and P-Type B. vulgaris

**Figure 3:**
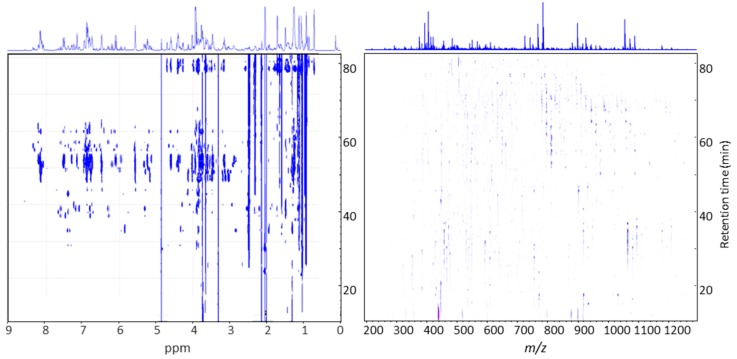
A 2D visualization of the time slice LC-SPE-NMR/MS data illustrated in the example of the saponin enriched extract of the G-type *B. vulgaris*.

In this experiment 1D proton NMR spectra were measured from extracts of post column enriched SPE fractions that have been collected at 1 min intervals during the entire LC separation along with mass spectrometric data obtained from a parallel detection using a flow splitter. A 2D visualization of the time slice LC-SPE-NMR/MS data is illustrated in Figure 3 in the example of the saponin enriched extract of the G-type *B. vulgaris*.

**Figure 4:**
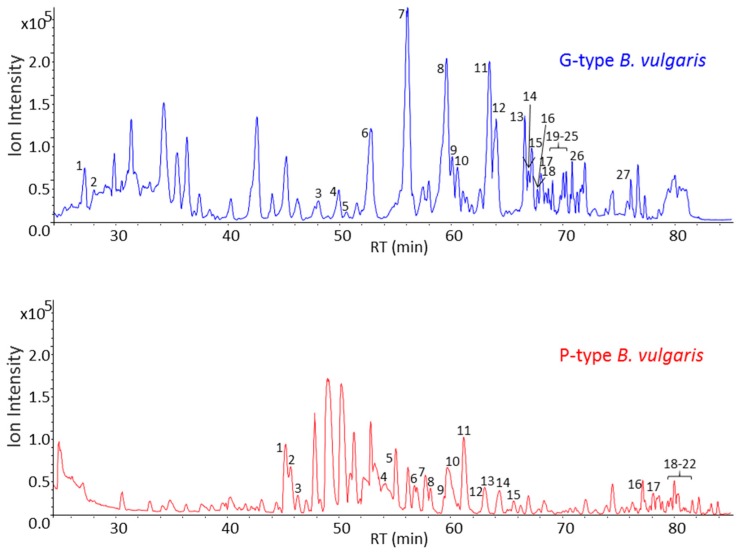
LC-MS base peak chromatogram (BPC) of saponin enriched extracts of the G- and P-type *B. vulgaris* obtained from the time slice LC-SPE-NMR/MS experiment. Peak numbers correspond to the numbering of tentatively identified saponins listed in Table 1 and Table 2 for G- and P-type plants, respectively.

The data acquired on saponin enriched extracts of G- and P-type plants were evaluated for the content of saponins. Visual inspection of the mass spectral data and corresponding NMR data measured on the same chromatographic interval resulted in 27 and 22 peaks from G- and P-type plants, respectively, that were tentatively identified as being saponins (Table 1 and Table 2). The time slice LC-SPE-NMR/MS data of the G-type plant was more complex and relative intensities of saponins were more abundant when compared to the P-type plant (Figure 4). Tentatively identified saponin peaks were characterized by their mass spectral fragmentation patterns that illustrated loss of adduct ions corresponding to sugar moieties including hexose (162 Da), methylpentose (146 Da), and pentose (132 Da).

The majority of saponins (17 out of 27) detected from the G-type plant contained only hexose moieties, whereas the number of saponins decorated with only hexose moieties in the P-type plant was found to be much less (seven out of 22). Moreover, most of the saponins of the P-type plant (14 out of 22) contained two types of sugars, methylpentose and hexose. Only one pentose containing saponin peak was detected from each plant type. The number of sugar moieties attached to the triterpenoid aglycone varied from 2 to 5 for both plants. The majority of saponins contained three sugar moieties. A total of 12 P-type and 16 G-type tentatively identified saponins contained three sugar moieties.

Mass spectral fragmentation patterns suggest that all 49 saponin peaks detected from both plants originate from eight different triterpenoid aglycones with different MW. The MW of all aglycones and mass spectral fragmentations that showed loss of sugar moieties matched with an observed MW of the corresponding saponin peak. A total of 10 saponins detected from the G-type plant corresponded to an aglycone with a MW of 472 Da, whereas nine P-type saponins were derived from an aglycone with a MW of 448 Da. In total, four aglycones with MW of 448, 458, 472, and 478, were common for both types of plants. G-type aglycones also included MW of 456 and 504 Da, and aglycones with MW of 464 and 474 Da were exclusively detected from the P-type plant. Tentatively characterized triterpenoid aglycones with MW of 456 and 472 Da correspond to the oleanolic acid and hederagenin, respectively, which were both identified from the G-type *B. vulgaris* in a previous study [19]. Other aglycones from *B. vulgaris* plants were found for the first time in this study. Despite the fact that previous studies performed on *B. vulgaris* have suggested the presence of saponins in P-type plants, no structural information about aglycones and their decoration by sugar moieties were available [2].

The 1D proton NMR spectra that represented a fingerprint of an entire eluent corresponding to a 1 min chromatographic interval further assisted in the characterization of tentatively identified saponins. Due to the complexity of the sample matrix and the similar physico-chemical properties of saponins, the resolution of the applied LC was not sufficient to separate some of saponins. Accordingly, the NMR spectra for some of the chromatographic intervals represent NMR signals of more than one saponin. In addition to the spectral complexity, the low S/N ratio of the NMR signals obtained from the time slice LC-SPE-NMR/MS experiment makes structure elucidation of saponins very challenging. Consequently, tentative identification of saponins was based on both their characteristic mass spectral fragmentation patterns and NMR features. One of the most characteristic NMR signals corresponding to saponins are the doublets derived from the anomeric protons of the sugar moieties attached to the triterpenoid aglycone. Despite the spectral complexity, it was possible to identify anomeric proton doublets for all tentatively characterized saponins. Most of these doublets were in the chemical shift range of 4.13–4.61 ppm with a spin-spin coupling constant of 7.8 Hz for both types of plants. Saponins that contained methylpentose moieties depicted doublets between 5.09–5.60 ppm with a spin-spin coupling constant of 7.8–8.2 Hz. The later finding is in agreement with a previously published study that showed similar chemical shifts for the methylpentose moieties of saponins [20]. It is worth to mention that spin-spin coupling constants found for all anomeric doublets suggest exclusively the β-configuration of the sugar moieties. This is in good agreement with the literature [19,21].

As mentioned previously, one of the main differences between the 1D proton NMR spectra of the G- and P-type *B. vulgaris* plants is a triplet, in the range of 5.2–5.3 ppm, which is absent in the P-type plants. This triplet corresponds to the methine group of the double bond at C12 position in the triterpenoid aglycone. Indeed the NMR data obtained from the time slice experiment showed triplet signals between 5.20–5.25 ppm, with a spin-spin coupling constant of 3.4 Hz, but only for the G-type saponins. This finding indicates that P-type saponins are not derived from β-amyrin, α-amyrin or other similar triterpenoid aglycones that possess a double bond at the C12 position. Our previous study performed on identification of genes involved in biosynthesis of saponins also confirmed that P-type *B. vulgaris* produces lupeol and lupeol derived aglycones, while G-type *B. vulgaris* accumulates mainly β-amyrin derived aglycones [10].

Figure S1 illustrates the mass spectral fragmentation patterns and 1D proton NMR spectra of some tentatively identified saponins from the G- and P-type *B. vulgaris*. The mass spectrum of the P-type saponin, which elutes at 78.4 min, exhibits a MW of 959 Da and a loss of 3 hexose moieties. Subsequently a MW of the aglycone was identified as 474 Da (Figure S1A). The NMR spectrum of the same saponin peak shows 5 singlets in the range of 0.7–1.7 ppm, which suggest the presence of 5 methyl groups on the triterpenoid aglycone. Three anomeric doublets corresponding to three hexose moieties were detected at 4.27 and 4.41 ppm with a spin-spin coupling constant of 7.8 Hz. In contrast to most G-type saponins, this peak did not possess a triplet around 5.2 which is supporting evidence for P-type saponins not being derived from β-amyrin or similar aglycones. The second chromatographic interval, at the retention time (RT) range of 79–80 min, for the P-type plant, suggests elution of at least two different saponins (Supplementary Figure S1B). The mass spectral fragmentation pattern that corresponds to the peak at the RT of 79.3 min has a molecular ion with a mass of 1105 Da and shows a subsequent loss of 4 hexose moieties. The corresponding aglycone ion is detected at *m/z* 457 which suggest that this saponin is based on an aglycone with MW of 458 Da. The mass spectral fragmentation pattern of the peak at RT of 79.7 min, from the same interval, reveals a loss of one methylpentose and three hexose moieties and a MW of the corresponding aglycone of 474 Da. The NMR data measured for this interval was also complex and showed 8 different doublets in the range of 4.27–5.60 ppm, with a spin-spin coupling constant of 7.8 or 8.2 Hz. Although it is difficult to quantify relative ratios of singlets, due to the complexity of the spectra, the five most intense singlets were observed in the range of 0.7–1.7 ppm. In addition to the spectral complexities observed at the aliphatic and anomeric regions, the complexity of the carbohydrate region, around 3.12–3.95 ppm, suggests co-elution of more than one type of saponin at this chromatographic interval. In contrast, the P-type saponins eluting in the RT range of 80.0–81.0 min have notably big differences in their relative concentrations, with the peak eluting at 80.4 min for the most dominant; accordingly the NMR spectra was less complex (Figure S1C). The tentative saponin peak eluting at 80.4 min possessed three hexose moieties, an aglycone with MW of 458 Da, and its molecular ion was detected at 943 *m/z*. A number of sugar moieties was confirmed from the 1D proton NMR data, which showed three distinct doublets at 4.27, 4.34, and 4.41 ppm, with a spin-spin coupling constant of 7.8 Hz. In addition to this, five singlets, between 0.7–1.7 ppm, that are consistently detected for P-type saponins were also detected for this tentative saponin.

**Figure 5:**
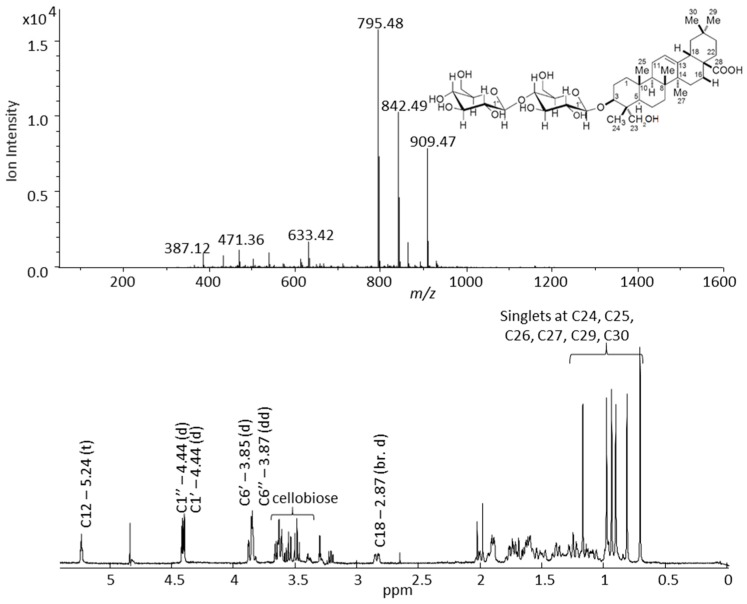
Negative mode mass spectral fragmentation pattern (upper figure) and 1D proton NMR spectrum (lower figure) of hederagenin cellobioside (peak 26 in Table 1) measured in an LC-SPE-NMR/MS experiment performed on a saponin enriched extract of G-type *B. vulgaris*.

**Figure 6:**
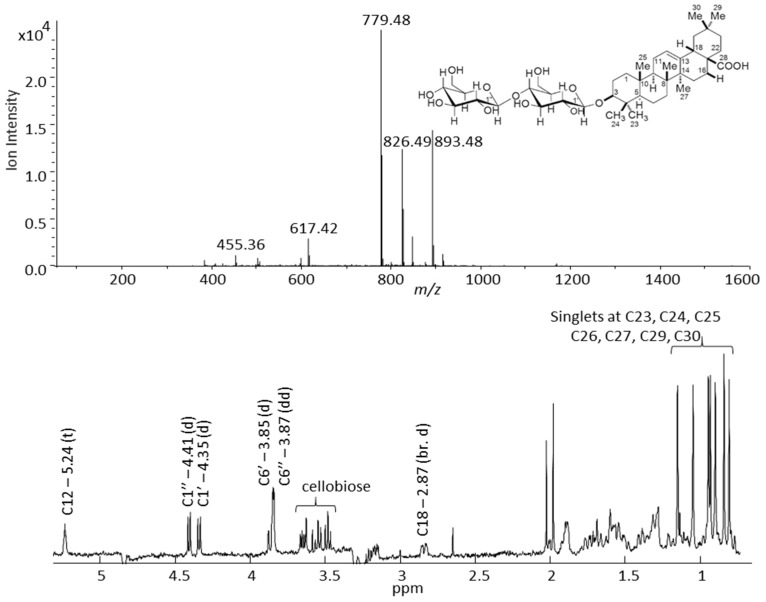
Negative mode mass spectral fragmentation pattern (upper figure) and 1D proton NMR spectrum (lower figure) of oleanolic acid cellobioside (peak 27 in Table 1) measured in an LC-SPE-NMR/MS experiment performed on a saponin enriched extract of G-type *B. vulgaris*.

Examples of mass spectral fragmentation patterns and NMR data for three tentatively identified G-type saponins are shown in Figure S1D–F. The G-type saponin, which eluted at the RT of 49.5 min, show a loss of three sugar moieties, one methylpentose and two hexoses, and its molecular ion and aglycone masses were 974 and 504 Da, respectively. The corresponding NMR data confirmed three doublets, at 4.27, 4.41, and 4.42 ppm, with spin-spin coupling constant of 7.8 Hz, a triplet at 5.25 ppm (*J* = 3.4 Hz) which is characteristic for the G-type saponins, and five singlets in the aliphatic region. Two other G-type saponins eluted at RT of 63.4 and 64.0 min and possessed aglycones with MWs of 472 and 458 Da, respectively, and both were decorated with three hexose moieties (Figure S1E,F). The two major insecticides [3,4] found in the G-type *B. vulgaris*, hederagenin cellobioside and oleanolic acid cellobioside, eluted at 72.8 and 75.8 min, respectively, and their mass spectral fragmentation patterns and 1D proton NMR spectra are shown in Figure 5 and Figure 6. The mass spectra and NMR data obtained from the time slice LC-NMR experiment for these two saponins were compared with the data reported in the literature and were unequivocally identified [3,4,19]. The mass spectrum of hederagenin cellobioside shows a molecular ion at *m/z* 795 that corresponds to the [M − H]^−^ anion, and a loss of two hexoses, in this case glucose moieties. The *m/z* ions at 633 and 471 correspond to [M – H − glucose]^−^ and [M − H − 2 × glucose]^−^ anions, respectively. Moreover, the 1D proton NMR data of hederagenin cellobioside confirmed the presence of 6 singlets derived from methyl groups of the hederagenin at C24, C25, C26, C27, C29, and C30 between 0.7–1.25 ppm. The doublets of two anomeric protons were detected at 4.41 and 4.42 ppm, with a coupling constant of 7.8 Hz, and the proton of C12 double bond was represented by a triplet at 5.24 ppm (*J* = 3.4 Hz). In addition, the proton at the tertiary carbon, C18, of hederagenin, and two methylene groups of glucose moieties were detected at 2.85 (dd, *J* = 4.3 and 14.2 Hz) and 3.86 (dd) ppm, respectively. Similarly, oleanolic acid cellobioside was identified and its mass spectral and NMR data matched with the data reported in the literature.

### 2.3. GC-MS Analysis of Saponin Enriched Extracts of G- and P-Types B. vulgaris 

**Figure 7:**
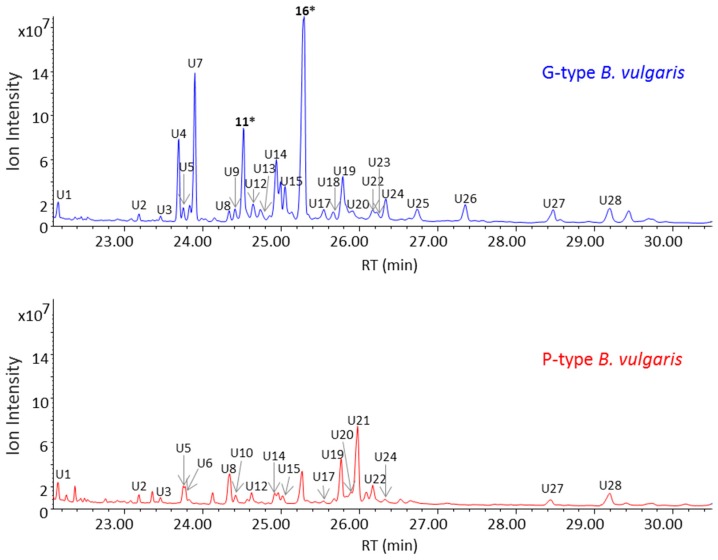
GC-MS total ion current (TIC) chromatograms of saponin enriched extracts of the G- and P-type *B. vulgaris* after acidic hydrolysis of the extracts which allowed cleave off sugar moieties attached to aglycones. A total of 28 peaks were detected from both plants. Electron impact-mass spectrum (EI-MS) fragmentation patterns of all 28 peaks are illustrated in Supplementary Figure S2. * Peak 11 and 16 were identified as oleanolic acid and hederagenin, respectively, using authentic standards of compounds.

Prior to screening of a triterpenoid aglycone pool of G- and P-type *B. vulgaris*, saponin enriched extracts were hydrolyzed using hydrochloric acid, then derivatized using trimethylsilyl cyanide and analysed by GC-MS. Total ion current (TIC) chromatograms of G- and P-type plants are shown in Figure 7. A total of 28 peaks were detected from both plants. As was also the case for the LC-MS data, the GC-MS data of the hydrolysed G-type plant was more complex, with additional and higher abundant peaks when compared to the P-type plant. Despite the fact that the majority of peaks were common for both plants, few peaks were exclusively present in only one type of plant. The G-type plant contained U4, U7, U9, and U13 while U10 and U21 were present only in P-type. Two major peaks that differ between the GC-MS profiles of G- and P-type plants were oleanolic acid (peak 11) and hederagenin (peak 16) which are only present in the G-type. These two peaks were the only ones identified at level 1 [22] using authentic standards of triterpenoid aglycones. This finding is in agreement with the LC-MS and 1D proton NMR data described earlier that show the presence of oleanolic acid cellobioside and hederagenin cellobioside from the G-type *B. vulgaris*.

EI-MS of all GC-MS peaks detected from the hydrolysed G- and P-type plants (Figure S2) were compared against authentic standards of triterpenes and only few peaks matched with a similarity of 75%–95%. The EI-MS spectrum of an unknown peak U9, which is exclusively found in G-type, exhibits 84% similarity with the mass spectrum of echinocystic acid, which is a β-amyrin derived triterpene. Despite the fact that the observed spectral similarity was relatively high, intensities of some *m*/*z* ions and retention indices differed significantly. The latter suggests that U9 is most probably not echinocystic acid, but probably a structurally similar triterpene. Two other G-type peaks, U25 and U26, eluting at late RT, 26.7 and 27.3 min, were tentatively identified as two different TMS derivatives of quillaic acid, a β-amyrin derived triterpene, which possesses two hydroxyl, one carboxylic acid, and one aldehyde functional groups. Moreover, an unknown peak observed only in the P-type plant, U21, displayed a similar but not identical EI-MS pattern (74%) to betulinic acid, a lupeol derived triterpene. Notable differences in relative intensities of *m*/*z* ions, including 175, 189, 292, and 320. In addition, GC-MS peaks which are detected exclusively in G-type plant, including U7, U13, U18, and U23, matched with EI-MS patterns of protopanaxatriol, caulophyllogenin, madecassic acid, and 6-β-maslinic acid, respectively, with at least 75% similarity. A common GC-MS peak for both plants, U19, possess similar (77%) EI-MS pattern as betulin, although it contains relatively abundant unique ions at *m*/*z* 473 and 512. A total of 10 out of 28 GC-MS peaks detected from both plants depicted EI-MS similarity of at least 75% when compared to an in-house developed library. However, authentic standards and/or 1D and 2D NMR data based comparison will be required prior to a level 1 identification.

## 3. Experimental Section

### 3.1. Plant Materials and Metabolite Extraction

Fresh leaves of 4–12 weeks old G- and P-type *Barbarea vulgaris* plants were used for the metabolite extraction. All plants were grown in a climate chamber as described previously [2]. Untargeted metabolite analysis using GC-MS, LC-MS, and NMR was performed on a global metabolite extract based on 85% methanol. Ten mg of fresh leave disks were quenched by rapid cooling using liquid nitrogen, transferred into 2.0 mL Eppendorf tubes, and extracted for 5 min with 0.8 mL 85% methanol at 100 °C by mixing at 1400 rpm in a thermomixer (Eppendorf, Hørsholm, Denmark). After this, the extracts were cooled on ice and centrifuged at 16,000 *g* for 3 min in order to obtain a clear supernatant for LC-MS and NMR analysis. Prior to GC-MS analysis, the methanol extract was hydrolysed as previously described [23], except the final solvent, diethyl ether, was replaced with ethyl acetate in a current study. It is worth to mention that residual acid hampers trimethylsilyl derivatization, so the ethyl acetate fraction must be washed with water.

Saponin enriched extracts of G- and P-type *B. vulgaris* plants were obtained from methanol extraction and enrichment of the saponin fraction was made by using solid phase extraction (SPE) cartridges. The freeze dried leaves of the plants were soaked in 55% ethanol (1:10 wt/vol) and boiled in a water bath for 5 min, followed by 10 min of extraction in an ultrasonic bath at 50 °C. The obtained extracts were filtered, dried under reduced pressure and re-dissolved in 30% methanol in a 3:1 vol/vol ratio between initial extract and methanol. Then, 60 mL of the 30% methanol extract was passed through a Strata C18 SPE cartridge (10 g, 60 mL) (Phenomenex ApS, Værløse, Denmark) which was pre-conditioned with 30% methanol and fraction 1 (30% methanol fraction) was obtained. Then, the same cartridge was flushed with 60 mL of 90% methanol which resulted in fraction 2 (90% methanol fraction), and finally 60 mL of 100% methanol (fraction 3) recovered the most non-polar metabolites of the plant extract. Fractions 1–3 were concentrated by drying using ScanVac (Labogene, Lynge, Denmark), and re-dissolved in 50% methanol, in a volume-to-volume ratio of 4:1 between the initial extracts and 50% methanol. Then, all fractions were analysed by LC-MS, which showed that fraction 2 (90% methanol) contained the majority of saponins of the *B. vulgaris* leaves. 

### 3.2. GC-MS Analysis

A total of 50 μL final metabolite extracts, either the 85% methanol or saponin extract, were completely dried using the ScanVac, operating at 40 °C, into 150 μL glass inserts. Immediately after drying, insets were sealed with air tight magnetic lids into GC–MS vials and derivatized by addition of 40 μL trimethylsilyl cyanide (TMSCN) [13]. All steps involving sample derivatization and injection were automated using a Dual-Rail MultiPurpose Sampler (MPS) (Gerstel, Mülheim an der Ruhr, Germany). After reagent addition, the sample was transferred into the agitator of the MPS and incubated at 40 °C for 40 min at 750 rpm. This procedure ensured precise derivatization time and reproducible sample injection. Immediately after derivatization, 1 μL of the derivatized sample was injected into a cooled injection system port (CIS4, Gerstel) in splitless mode. The septum purge flow and purge flow to split vent at 2.5 min after injection were set to 25 and 15 mL/min, respectively. Initial temperature of the CIS4 port was 120 °C, and heated at 5 °C/s to 320 °C (after 30 s of equilibrium time), where it was kept for 10 min. After heating, the CIS4 port was gradually cooled to 250 °C at 5 °C/s, and this temperature was kept constant during the entire run. The GC-MS consisted of an Agilent 7890A GC and an Agilent 5975C series MSD (Agilent Technologies, Glostrup, Denmark). GC separation was performed on an Agilent HP-5MS column (30 m × 250 μm × 0.25 μm) by using hydrogen carrier gas at a constant flow rate of 1.2 mL/min. The GC oven temperature program was as follows: initial temperature, 40 °C; heating rate, 12.0 °C·min^−1^; end temperature, 300 °C; hold time, 8.0 min; and post-run time, 5 min at 40 °C. Mass spectra were recorded in the *m*/*z* range of 50–600 with a scanning frequency of 2.3 scan/s, and the MS detector was switched off during the 8.5 min solvent delay time. The transfer line, ion source, and quadrupole temperatures were set to 280, 230, and 150 °C, respectively. The mass spectrometer was tuned according to manufacturer recommendations by using perfluorotributylamine. The MPS and GC-MS was controlled using vendor software Maestro (Gerstel). A blank sample containing only derivatization reagent was run in order to monitor reagent derived and column derived non-sample related peaks. An alkane mixture standard sample (all even C_10_-C_40_ alkanes at 50 mg/L in hexane) was used prior to calculate retention indices. 

### 3.3. NMR Analysis

An aliquot (300 μL) of 85% metabolite extracts from G- and P-type *B. vulgaris* plant leaves were completely dried in 1.5 mL glass vials and re-dissolved in 500 μL methanol-*d*_4_ (99.8%) which contained 5 μL of 20 mg·mL^−1^ TSP in deuterated water. A total of 10 G-type, 10 P-type biological replicates, and three 50% G-type and 50% P-type mixture samples were investigated. 1D proton NMR spectra were recorded using an Avance DSX 500 NMR spectrometer (11.7 Tesla) (Bruker Biospin GmbH, Rheinstetten, Germany) operating at 500.13 MHz, and equipped with a BBI probe for 5 mm (o.d.) sample tubes. Data acquisition for all the samples was automated using the IconNMR automation software (version 3.5, Bruker Biospin GmbH, Rheinstetten, Germany) and each sample was automatically tuned, matched and shimmed. A total of 512 scans were recorded at room temperature with a relaxation delay of 5 s, a sweep width of 20 ppm, and 32 k complex data points. The obtained spectra were referenced to the 3-(trimethylsilyl)-propionic acid-*d*_4_ (TSP) peak at 0.00 ppm. Raw NMR data of 23 samples were imported into MATLAB (Version 7.13.0.564, R2011b, MathWorks Inc., Natick, MA, USA) after baseline and phase correction using TopSpin (version 2.1 Bruker BioSpin). The multivariate data analysis included interval based extended canonical variate analysis (iECVA) which was performed using in-house MATLAB scripts.

### 3.4. LC-SPE-NMR/MS Analysis

The system consisted of an Agilent 1200 HPLC system equipped with a quaternary pump, auto sampler, diode array detector, a Bruker/Spark Prospekt 2 LC-SPE system (Spark, Emmen, The Netherlands) and a MicrOTOF mass spectrometer (Bruker Daltonics, Bremen, Germany) connected via a Bruker NMR MS Interface (BNMI-HP). MS spectra, in negative mode, were acquired between *m/z* 50 and 1600. The calibration was done with a 20 mM lithium formate automatically which is introduced at the beginning of each chromatographic run. Separation was done on a Prodigy LC column (ODS3, 5 µm particle size, and 250 mm × 4.6 mm; Phenomenex, Aschaffenburg, Germany). The chromatographic conditions were as following; a flow rate of 0.5 mL/min starting with a solvent composition of 90% A (H_2_O 0.1% formic acid-*d*_2_) and 10% B (acetonitrile 0.1% formic acid-*d*_2_) with a linear gradient to 70% A at 60 min, followed by another linear gradient to 10% A at 80 min. Starting at 15 min the eluent was collected on SPE cartridges. For this trapping process a makeup-flow of 1.5 mL·min^−1^ (mobile phase A) was added to the eluent before it passed through the SPE cartridges in order to increase the retention of analytes on the cartridges. For a time slice LC-SPE-NMR/MS experiment, 1 min period of the chromatograms were collected on a cartridge, before the system automatically changed to the next cartridge. Therefore the content of one cartridge contains the compounds eluted during this period of time from the column. For the trapping 10 mm × 2 mm SPE cartridges filled with GP resin were used.

Prior to NMR measurements of SPE cartridge eluents, samples were dried with a stream of nitrogen, the fraction from each cartridge being eluted using a total of 250 µL deuterated methanol. The sample was eluted directly into a CryoFit with a 30 µL flow cell placed in a TCI CryoProbe Prodigy with a Bruker Avance III 500 MHz. NMR spectra were acquired with a relaxation delay of 5 s and a sweep width of 10 ppm, and 16 k complex data points. A 1D-WET multiple solvent suppression on two solvent resonances was used (residual OH methanol signal). 128 transients were acquired. ^13^C satellites of the methanol were selectively decoupled with a low power continuous wave decoupling using the 2nd channel of the spectrometer. After multiplication of the resulting free induction decay with an exponential function leading to a peak broadening of 1.0 Hz, Fourier transformation was performed followed by phase and baseline correction leading to a single NMR spectrum. All spectra were combined into a pseudo-2D file which shows NMR spectrum against chromatographic retention time.

## 4. Conclusions

This study has shown how integrated and hyphenated analytical platforms can be used to elucidate the diversity of saponins (aglycone and sugar decoration) from plant tissues. The LC-SPE-NMR/MS and GC-MS study revealed a total of eight different triterpenoid aglycones with different MWs. Four aglycones with MW of 448, 458, 472, and 478 were found to be common for both types of plants. Two with MWs of 456 and 504 Da was found to be exclusive to G-type plants and two with MW of 464 and 474 Da were exclusive detected from the P-type plant. The average glycosylation of the aglycones were three sugar residues and were exclusively found in β-anomer configuration. The study suggests that P-type *B. vulgaris* produces lupeol and lupeol derived aglycones, while G-type *B. vulgaris* produces mainly β-amyrin derived aglycones, in agreement with previous studies. GC-MS of saponin enriched extracts of G- and P-types *B. vulgaris* confirmed the identity of the saponin aglycones. The G-type extracts were found to be much more complex, including the two major saponin aglycons, oleanolic acid and hederagenin, which are only present in G-type. These two peaks were the only ones with identities confirmed at level 1 [22], using authentic standards of triterpenoid aglycones. In order to elucidate structures of G- and P-type saponins, observed from the LC-SPE-NMR/MS and GC-MS analysis, we are currently in progress with LC fractionation of saponin enriched extracts, from both plants, prior to 2D NMR and GC-MS analysis of pure saponin fractions. 

## Figures and Tables

**Table 1:** List of tentatively annotated triterpenoid saponins from the time slice LC-SPE-NMR/MS experiment performed on saponin enriched extract of the glabrous (G) type *B. vulgaris*.

No.	RT (min)	Aglycone (Da) ^A^	Sugar Moieties ^B^	Mass Spectra Fragmentation ^C^	Anomeric Protons (ppm) ^D^
1	27.3	448	2 × Methylpentose 3 × Hexose	[M_1225_ − H − 162 − 146 − 162 − 146 − 162]^−^ = 447	4.13 (d, *J* = 7.8) 4.17 (d, *J* = 7.8) 4.29 (d, *J* = 7.8)
2	27.7	478	2 × Methylpentose 1 × Pentose 2 × Hexose	[M_1225_ − H − 162 − 132 – 162 − 146 − 146]^−^ = 477	
3	49.5	504	1 × Methylpentose 2 × Hexose	[M_973_ − H − 146 − 162 − 162]^−^ = 503	4.21 (d, *J* = 7.8) 4.41 (d, *J* = 7.8) 4.42 (d, *J* = 7.8) 5.25 * (t, *J* = 3.4)
4	50.5	504	1 × Methylpentose 2 × Hexose	[M_973_ − H − 146 − 162 − 162]^−^ = 503	4.21 (d, *J* = 7.8) 4.41 (d, *J* = 7.8) 4.42 (d, *J* = 7.8) 5.25 * (t, *J* = 3.4)
5	51.2	504	1 × Methylpentose 2 × Hexose	[M_973_ − H − 162 − 146 − 162]^−^ = 503	4.21 (d, *J* = 7.8) 4.39 (d, *J* = 7.8) 5.25 * (t, *J* = 3.4)
6	52.5	649 **	3 × Hexose	[M_1135_ − H − 162 − 162 − 162]^−^ = 649	4.41 (d, *J* = 8.0) 4.43 (d, *J* = 8.0) 5.55 (d, *J* = 8.2)
7	56.2	649 **	2 × Hexose	[M_973_ − H − 162 − 162]^−^ = 649	4.41 (d, *J* = 8.0) 5.52 (d, *J* = 8.2)
8	59.5	649 **	2 × Hexose	[M_973_ − H − 162 − 162]^−^ = 649	4.41 (d, *J* = 8.0) 5.52 (d, *J* = 8.2)
9	60.6	472	4 × Hexose	[M_1119_ − H − 162 − 162 − 162 − 162]^−^ = 471	4.34 (d, *J* = 7.8) 4.41 (d, *J* = 8.0) 4.43 (d, *J* = 7.8) 5.55 (d, *J* = 8.0)
10	61.2	633	3 × Hexose	[M_1119_ − H − 162 − 162 − 162]^−^ = 633	4.34 (d, *J* = 7.8) 4.41 (d, *J* = 8.0) 4.43 (d, *J* = 7.8) 5.55 (d, *J* = 8.0)
11	63.4	472	3 × Hexose	[M_957_ − H − 162 − 162 − 162]^−^ = 471	4.27 (d, *J* = 7.8) 4.34 (d, *J* = 7.8) 4.41 (d, *J* = 7.8) 5.24 * (t, *J* = 3.4) 5.53 (d, *J* = 8.0)
12	64.0	458	3 × Hexose	[M_943_ − H − 162 − 162 − 162]^−^ = 457	4.27 (d, *J* = 7.8) 4.34 (d, *J* = 7.8) 4.41 (d, *J* = 7.8)
13	66.2	619 **	2 × Hexose	[M_943_ − H − 162 − 162]^−^ = 457	4.17 (d, *J* = 7.8) 4.27 (d, *J* = 7.8) 4.34 (d, *J* = 7.8) 4.35 (d, *J* = 7.8) 4.41 (d, *J* = 7.8) 5.20 * (t, *J* = 3.4)
14	66.6	472	3 × Hexose	[M_957_ − H − 162 − 162 − 162]^−^ = 471	
15	67.2	458	3 × Hexose	[M_943_ – H − 162 − 162 − 162]^−^ = 457	4.18 (d, *J* = 7.8) 4.26 (d, *J* = 7.8) 4.35 (d, *J* = 7.8) 4.41 (d, *J* = 7.8) 5.25 * (t, *J* = 3.4) 5.38 (d, *J* = 8.3)
16	67.6	472	4 × Hexose	[M_1119_ − H − 162 − 162 − 162 − 162]^−^ = 471	
17	68.2	458	3 × Hexose	[M_943_ − H − 162 − 162 − 162]^−^ = 457	4.18 (d, *J* = 7.8) 4.22 (d, *J* = 7.8) 4.27 (d, *J* = 6.0) 4.30 (d, *J* = 6.0) 4.35 (d, *J* = 6.9) 4.41 (d, *J* = 7.8) 5.20 * (t, *J* = 3.4) 5.25 * (t, *J* = 3.4)
18	68.3	472	3 × Hexose	[M_957_ − H − 162 – 162 − 162]^−^ = 471	
19	68.5	472	2 × Methylpentose 1 × Hexose	[M_925_ − H − 146 − 146 − 162]^−^ = 471	
20	69.1	472	1 × Methylpentose 2 × Hexose	[M_941_ − H − 146 − 162 − 162]^−^ = 471	4.16 (d, *J* = 6.6) 4.35 (d, *J* = 7.8) 4.41 (d, *J* = 7.8) 5.20 * (t, *J* = 3.4) 5.25 * (t, *J* = 3.4)
21	69.5	458	3 × Hexose	[M_943_ − H − 162 − 162 − 162]^−^ = 457	
22	69.7	456	3 × Hexose	[M_941_ − H − 162 − 162 − 162]^−^ = 455	
23	70.2	472	1 × Methylpentose 2 × Hexose	[M_941_ − H − 146 − 162 − 162]^−^ = 471	4.35 (d, *J* = 7.8) 4.41 (d, *J* = 7.8) 5.25 * (t, *J* = 3.4) 5.30 * (t, *J* = 3.4)
24	70.4	456	3 × Hexose	[M_941_ − H − 162 − 162 − 162]^−^ = 455	
25	70.4	472	2 × Methylpentose 1 × Hexose	[M_925_ − H − 146 − 162 − 146]^−^ = 471	
26	72.8	472 Hederagenin cellobioside	2 × Hexose	[M_795_ – H − 162 − 162]^−^ = 471	4.41 (d, *J* = 7.8) 4.42 (d, *J* = 7.8) 5.24 * (t, *J* = 3.4)
27	75.8	456 Oleanolic ac. cellobioside	2 × Hexose	[M_779_ − H − 162 − 162]^−^ = 455	4.34 (d, *J* = 7.8) 4.41 (d, *J* = 7.8) 5.23 * (t, *J* = 3.4)

^A^ A molecular weight of a saponin aglycone observed from the mass spectral fragmentation pattern of a corresponding peak. ^B^ Tentatively identified number and type of sugar moieties. ^C^ Mass spectral fragmentation patterns illustrated a loss of sugar moieties including hexose (162 Da), methyl-pentose (146 Da), and pentose (132 Da) that are possibly attached to the corresponding triterpenoid aglycone. Adduct ions of sugar moieties correspond to the molecular weights of sugars minus a water molecule. ^D^ Chemical shift (ppm) and spin-spin coupling constants (Hz) of anomeric protons of possible sugar moieties. Note: LC-MS was performed in (-) negative mode and 1D proton NMR spectrum (number of scans = 128) with water suppression was recorded for each interval of 1 min during the entire run. * Triplet derived from the methine group at the C12 position of an aglycone (e.g., hederagenin and oleanolic acid). ** Loss of the last sugar moiety was not observed.

**Table 2:** List of tentatively annotated triterpenoid saponins from the time slice LC-SPE-NMR/MS experiment performed on saponin enriched extract of the pubescent (P) type *B. vulgaris*.

No.	RT (min)	Aglycone (Da) ^A^	Sugar Moieties ^B^	Fragmentation Pattern in Mass Spectra ^C^	Anomeric Protons (ppm) ^D^
1	45.2	448	5 × Hexose	[M_1257_ − H − 162 − 162 − 162 − 162 − 162]^−^ = 447	4.13 (d, *J* = 7.8) 5.20 (d, *J* = 7.8) 5.28 (d, *J* = 7.8)
2	45.7	464	3 × Methylpentose 2 × Hexose	[M_1225_ − H − 162 − 146 − 162 − 146 − 146]^−^ = 463	
3	46.4	478	1 × Pentose 1 × Hexose 1 × Methylpentose	[M_917_ − H − 132 − 162 − 146]^−^ = 477	4.13 (d, *J* = 7.8) 4.63 (d, *J* = 7.8) 5.17 (d, *J* = 7.8)
4	54.1	448	2 × Methylpentose 1 × Hexose	[M_901_ − H − 146 − 146 − 162]^−^ = 447	4.35 (d, *J* = 8.0) 4.39 (d, *J* = 8.0) 4.58 (d, *J* = 8.0) 4.60 (d, *J* = 8.0) 5.12 (d, *J* = 7.8) 5.23 (d, *J* = 7.8)
5	54.8	464	3 × Methylpentose	[M_901_ − H − 146 − 146 − 146]^−^ = 463	
6	56.8	448	2 × Methylpentose 1 × Hexose	[M_901_ − H − 146 − 146 − 162]^−^ = 447	4.04 (d, *J* = 8.0) 4.42 (d, *J* = 8.0) 4.75 (d, *J* = 7.8)
7	57.7	448	2 × Methylpentose 1 × Hexose	[M_901_ − H − 146 − 146 − 162]^−^ = 447	4.29 (d, *J* = 7.8) 4.43 (d, *J* = 7.8) 4.66 (d, *J* = 7.8)
8	58.4	609^*^	1 × Methylpentose 1 × Hexose	[M_917_ − H − 146 − 162]^−^ = 609	4.19 (d, *J* = 7.8) 4.31 (d, *J* = 7.8) 5.14 (d, *J* = 7.8)
9	59.6	448	1 × Methylpentose 2 × Hexose	[M_917_ − H − 162 − 162 − 146]^−^ = 447	4.19 (d, *J* = 7.8) 4.39 (d, *J* = 7.8) 4.53 (d, *J* = 7.8)
10	61.2	448	2 × Methylpentose 1 × Hexose	[M_901_ − H − 146 − 146 − 162]^−^ = 447	4.37 (d, *J* = 7.8) 4.42 (d, *J* = 8.0) 4.60 (d, *J* = 8.0) 4.62 (d, *J* = 8.0) 5.09 (d, *J* = 7.8) 5.20 (d, *J* = 7.8)
11	61.8	448	2 × Methylpentose 1 × Hexose	[M_901_ − H − 146 − 146 − 162]^−^ = 447	
12	62.7	472	2 × Hexose	[M_795_ − H − 162 − 162]^−^ = 471	4.19 (d, *J* = 7.8) 5.14 (d, *J* = 7.8)
13	63.1	448	2 × Methylpentose 1 × Hexose	[M_901_ − H − 146 − 146 − 162]^−^ = 447	4.42 (d, *J* = 8.0) 4.60 (d, *J* = 8.0) 4.62 (d, *J* = 8.0)
14	64.1	623^*^	1 × Methylpentose 1 × Hexose	[M_931_ − H − 146 − 162]^−^ = 623	4.15 (d, *J* = 8.0) 4.19 (d, *J* = 7.8) 4.62 (d, *J* = 8.0) 4.68 (d, *J* = 8.0) 5.13 (d, *J* = 7.8) 5.28 (d, *J* = 7.8) 5.57 (d, *J* = 8.0)
15	64.6	448	2 × Methylpentose 1 × Hexose	[M_901_ − H − 146 − 146 − 162]^−^ = 447	
16	77.4	474	4 × Hexose	[M_1121_ − H − 162 − 162 − 162 − 162 ]^−^ = 473	4.30 (d, *J* = 7.8) 4.40 (d, *J* = 7.8) 4.41 (d, *J* = 7.8) 4.71 (d, *J* = 8.0)
17	78.4	474	3 × Hexose	[M_959_ − H − 162 − 162 − 162 ]^−^ = 473	4.27 (d, *J* = 7.8) 4.41 (d, *J* = 7.8) 4.41 (d, *J* = 7.8)
18	79.1	458	4 × Hexose	[M_1105_ − H − 162 − 162 − 162 − 162]^−^ = 457	4.27 (d, *J* = 7.8) 4.34 (d, *J* = 7.8) 4.38 (d, *J* = 7.8) 4.39 (d, *J* = 7.8) 4.41(d, *J* = 7.8) 4.62 (d, *J* = 7.8) 5.53 (d, *J* = 8.2) 5.60 (d, *J* = 8.2)
19	79.4	472	4 × Hexose	[M_1119_ − H − 162 − 162 − 162 − 162 ]^−^ = 471	
20	79.8	474	1 × Methylpentose 3 × Hexose	[M_1105_ − H − 146 − 162 − 162 − 162 ]^−^ = 473	
21	80.4	458	4 × Hexose	[M_943_ − H − 162 − 162 − 162 ]^−^ = 457	4.27 (d, *J* = 7.8) 4.31 (d, *J* = 7.8) 4.34 (d, *J* = 7.8) 4.38 (d, *J* = 7.8) 4.39 (d, *J* = 7.8) 4.41 (d, *J* = 7.8) 4.61 (d, *J* = 7.8)
22	80.8	472	1 × Methylpentose 3 × Hexose	[M_1103_ − H − 162 − 162 − 146 − 162 ]^−^ = 471	

^A^ A molecular weight of a saponin aglycone observed from the mass spectral fragmentation pattern of a corresponding peak. ^B^ Tentatively identified number and type of sugar moieties. ^C^ Mass spectral fragmentation patterns illustrated a loss of sugar moieties including hexose (162 Da), methyl-pentose (146 Da), and pentose (132 Da) that are possibly attached to the corresponding triterpenoid aglycone. Adduct ions of sugar moieties correspond to the molecular weights of sugars minus a water molecule. ^D^ Chemical shift (ppm) and spin-spin coupling constants (Hz) of anomeric protons of possible sugar moieties. Note: LC-MS was performed in (-) negative mode and 1D proton NMR spectrum (number of scans = 128) with water suppression was recorded for each interval of 1 min during the entire run. * Loss of the last sugar moiety was not observed.

## Supplementary Materials

Supplementary materials can be accessed at: http://www.mdpi.com/1420-3049/21/12/1614/s1.

## References

[1] Augustin J.M., Kuzina V., Andersen S.B., Bak S. (2011). Molecular activities, biosynthesis and evolution of triterpenoid saponins. Phytochemistry.

[2] Kuzina V., Ekstrom C.T., Andersen S.B., Nielsen J.K., Olsen C.E., Bak S. (2009). Identification of Defense Compounds in *Barbarea vulgaris* against the Herbivore Phyllotreta nemorum by an Ecometabolomic Approach. Plant Physiol..

[3] Shinoda T., Nagao T., Nakayama M., Serizawa H., Koshioka M., Okabe H., Kawai A. (2002). Identification of a triterpenoid saponin from a crucifer, *Barbarea vulgaris*, as a feeding deterrent to the diamondback moth, Plutella xylostella. J. Chem. Ecol..

[4] Agerbirk N., Olsen C.E., Bibby B.M., Frandsen H.O., Brown L.D., Nielsen J.K., Renwick J.A.A. (2003). A saponin correlated with variable resistance of *Barbarea vulgaris* to the diamondback moth *Plutella xylostella*. J. Chem. Ecol..

[5] Di Fabio G., Romanucci V., de Marco A., Zarrelli A. (2014). Triterpenoids from Gymnema sylvestre and Their Pharmacological Activities. Molecules.

[6] Asl M.N., Hosseinzadeh H. (2008). Review of pharmacological effects of *Glycyrrhiza* sp. and its bioactive compounds. Phytother. Res..

[7] Liu J. (1995). Pharmacology of oleanolic acid and ursolic acid. J. Ethnopharmacol..

[8] Shah M.R., Ishtiaq, Hizbullah S.M., Habtemariam S., Zarrelli A., Muhammad A., Collina S., Khan I. (2016). Protein tyrosine phosphatase 1B inhibitors isolated from Artemisia roxburghiana. J. Enzym. Inhib. Med. Chem..

[9] Augustin J.M., Drok S., Shinoda T., Sanmiya K., Nielsen J.K., Khakimov B., Olsen C.E., Hansen E.H., Kuzina V., Ekstrom C.T. (2012). UDP-Glycosyltransferases from the UGT73C Subfamily in *Barbarea vulgaris* Catalyze Sapogenin 3-*O*-Glucosylation in Saponin-Mediated Insect Resistance. Plant Physiol..

[10] Khakimov B., Kuzina V., Erthmann P.O., Fukushima E.O., Augustin J.M., Olsen C.E., Scholtalbers J., Volpin H., Andersen S.B., Hauser T.P. (2015). Identification and genome organization of saponin pathway genes from a wild crucifer, and their use for transient production of saponins in Nicotiana benthamiana. Plant J..

[11] Geisler K., Hughes R.K., Sainsbury F., Lomonossoff G.P., Rejzek M., Fairhurst S., Olsen C.E., Motawia M.S., Melton R.E., Hemmings A.M. (2013). Biochemical analysis of a multifunctional cytochrome P450 (CYP51) enzyme required for synthesis of antimicrobial triterpenes in plants. Proc. Natl. Acad. Sci. USA.

[12] Madl T., Sterk H., Mittelbach M., Rechberger G.N. (2006). Tandem Mass Spectrometric Analysis of a Complex Triterpene Saponin Mixture of Chenopodium quinoa. J. Am. Soc. Mass Spectrom..

[13] Khakimov B., Motawia M.S., Bak S., Engelsen S.B. (2013). The use of trimethylsilyl cyanide derivatization for robust and broad-spectrum high-throughput gas chromatography-mass spectrometry based metabolomics. Anal. Bioanal. Chem..

[14] Khakimov B. (2013). Metabolomics and Bioactive Substances in Plants. Ph.D. Thesis.

[15] Harshman R.A. (1972). PARAFAC2: Mathematical and technical notes. UCLA Work. Pap. Phon..

[16] Bro R., Andersson C.A., Kiers H.A.L. (1999). PARAFAC2—Part II. Modeling chromatographic data with retention time shifts. J. Chemometr..

[17] Khakimov B., Amigo J.M., Bak S., Engelsen S.B. (2012). Plant metabolomics: Resolution and quantification of elusive peaks in liquid chromatography-mass spectrometry profiles of complex plant extracts using multi-way decomposition methods. J. Chromatogr. A.

[18] Nørgaard L., Bro R., Westad F., Engelsen S.B. (2006). A modification of canonical variates analysis to handle highly collinear multivariate data. J. Chemometr..

[19] Nielsen N.J., Nielsen J., Staerk D. (2010). New Resistance-Correlated Saponins from the Insect-Resistant Crucifer *Barbarea vulgaris*. J. Agric. Food Chem..

[20] Wan C.P., Yu Y.Y., Zhou S.R., Tian S.G., Cao S.W. (2011). Isolation and identification of phenolic compounds from Gynura divaricata leaves. Pharmacogn. Mag..

[21] Agrawal P.K. (2005). Assigning stereodiversity of the 27-Me group of furostane-type steroidal saponins via NMR chemical shifts. Steroids.

[22] Sumner L., Amberg A., Barrett D., Beale M., Beger R., Daykin C., Fan T., Fiehn O., Goodacre R., Griffin J. (2007). Proposed minimum reporting standards for chemical analysis. Metabolomics.

[23] Khakimov B., Mongi R.J., Sørensen K.M., Ndabikunze B.K., Chove B.E., Engelsen S.B. (2016). A comprehensive and comparative GC-MS metabolomics study of non-volatiles in Tanzanian grown mango, pineapple, jackfruit, baobab and tamarind fruits. Food Chem..

