# Supplementary material for: Screening for Triterpenoid Saponins in Plants Using Hyphenated Analytical Platforms
Source: Molecules. 2016 Nov 24;21(12):1614. doi: 10.3390/molecules21121614 (PMC6273444; doi:10.3390/molecules21121614)
Supplement: Supplementary file 1 [file molecules-21-01614-s001.pdf]

Supplementary Materials: Screening for Triterpenoid Saponins in Plants Using Hyphenated Analytical Platforms

Bekzod Khakimov, Li Hong Tseng, Markus Godeljohann, Søren Bak and Søren Balling Engelsen

A

№	RT (min)	Aglycone (Da)	Sugar Moieties	Fragmentation pattern in mass spectra	Anomeric protons (ppm)
P-type Peak 17	78.4	474	3 × Hexose	$[M_{959} – H –162 – 162 – 162]^{-}$= 473	4.27 (d, J = 7.8)<br>4.41 (d, J = 7.8)<br>4.41 (d, J = 7.8)

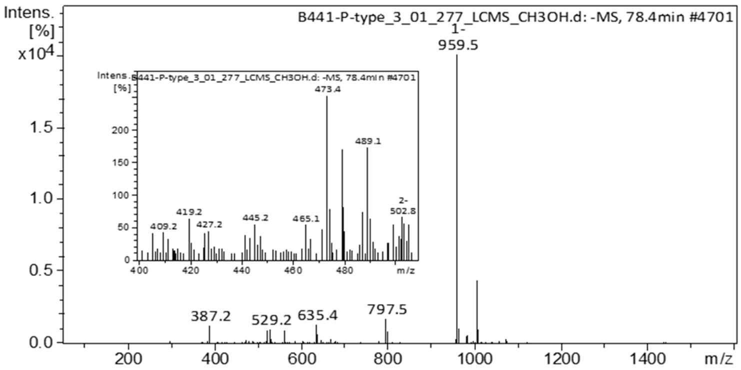

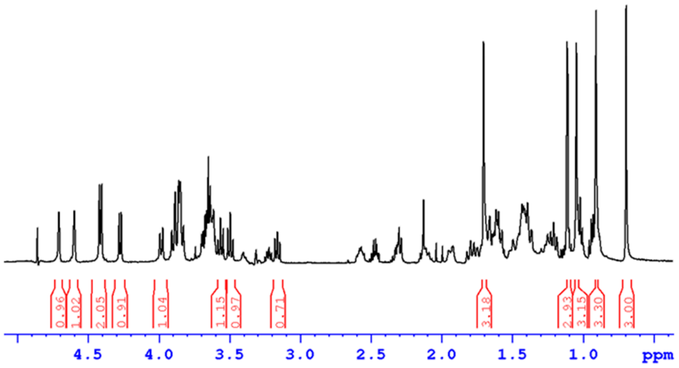

B

№	RT (min)	Aglycone (Da)	Sugar Moieties	Fragmentation pattern in mass spectra	Anomeric protons (ppm)
P-type Peak 18	79.1	458	4 × Hexose	$[M_{1105} – H – 162 – 162 – 162 – 162]^{-}$= 457	4.27 (d, J = 7.8)<br>4.34 (d, J = 7.8)<br>4.382 (d, J = 7.8)<br>4.386 (d, J = 7.8)<br>4.41(d, J = 7.8)<br>4.61 (d, J = 7.8)<br>5.53 (d, J = 8.2)<br>5.60 (d, J = 8.2)
P-type Peak 20	79.8	474	1 × Methylpentose<br>3 × Hexose	$[M_{1105} – H – 146 – 162 – 162 – 162]^{-}$= 473	

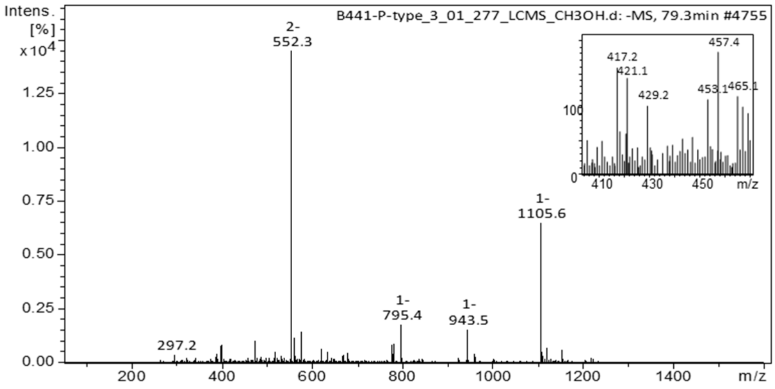

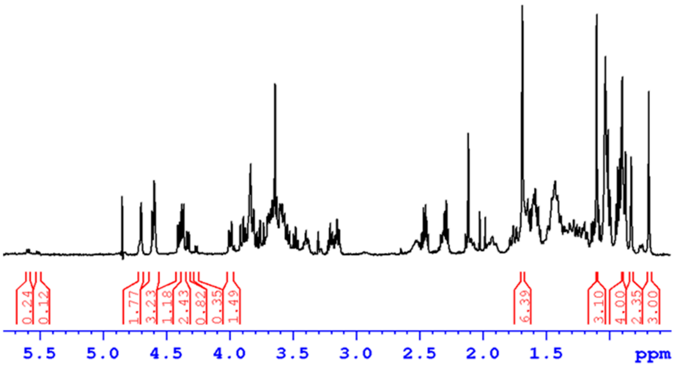

C

№	RT (min)	Aglycone (Da)	Sugar Moieties	Fragmentation pattern in mass spectra	Anomeric protons (ppm)
P-type Peak 21	80.4	458	4 × Hexose	$[M_{943} - H - 162 - 162 - 162]^{-} = 457$	4.27 (d, J = 7.8)<br>4.34 (d, J = 7.8)<br>4.41(d, J = 7.8)

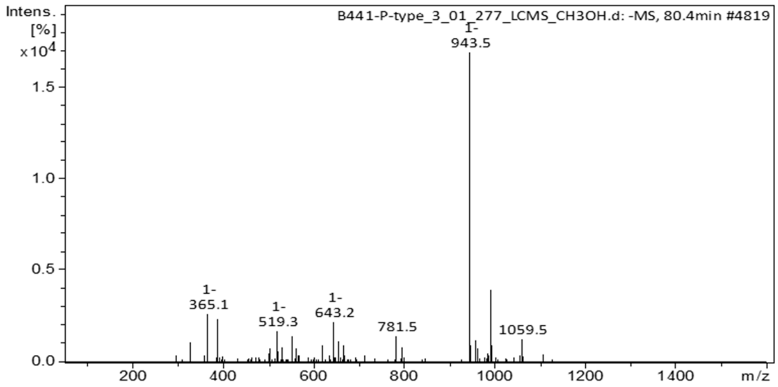

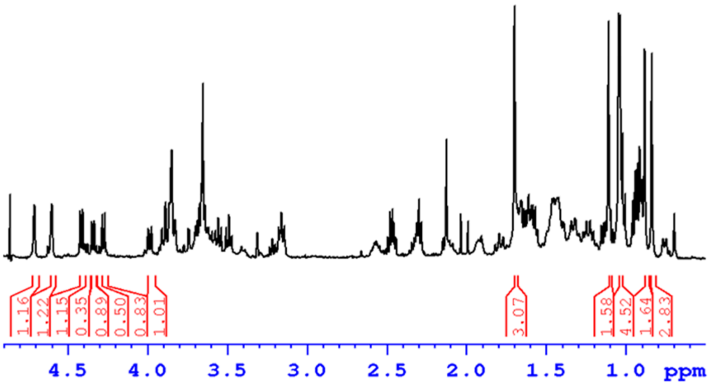

D

№	RT (min)	Aglycone (Da)	Sugar Moieties	Fragmentation pattern in mass spectra	Anomeric protons (ppm)
G-type Peak 3	49.5	504	1 × Methylpentose<br>2 × Hexose	$[M_{973} - H - 146 - 162 - 162]^{-} = 503$	4.21 (d, J = 7.8)<br>4.41 (d, J = 7.8)<br>4.42 (d, J = 7.8)<br>5.25* (t, J = 3.4)

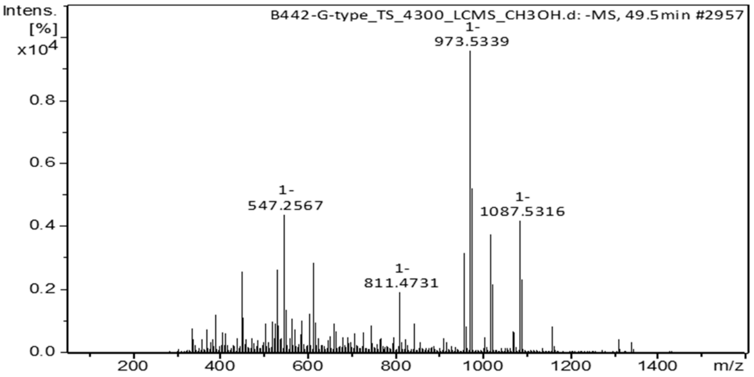

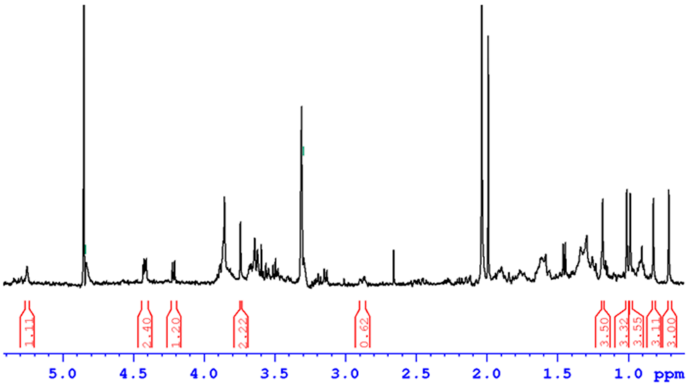

E

№	RT (min)	Aglycone (Da)	Sugar Moieties	Fragmentation pattern in mass spectra	Anomeric protons (ppm)
G-type Peak 11	63.4	472	3 × Hexose	$[M_{957} - H - 162 - 162 - 162]^{-} = 471$	4.27 (d, J = 7.8)<br>4.34 (d, J = 7.8)<br>4.41 (d, J = 7.8)<br>5.24* (t, J = 3.4)<br>5.53 (d, J = 8.0)

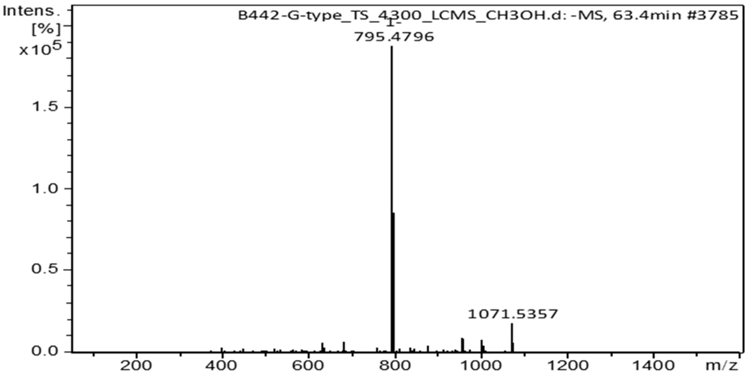

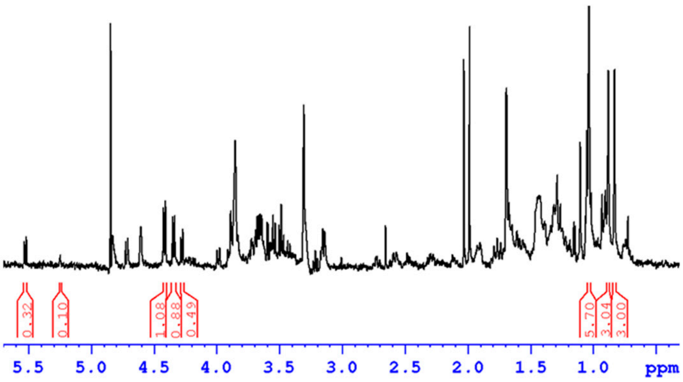

F

№	RT (min)	Aglycone (Da)	Sugar Moieties	Fragmentation pattern in mass spectra	Anomeric protons (ppm)
G-type Peak 12	64.0	458	3 × Hexose	$[M_{943} - H - 162 - 162 - 162]^{-} = 457$	4.27 (d, J = 7.8)<br>4.34 (d, J = 7.8)<br>4.41 (d, J = 7.8)

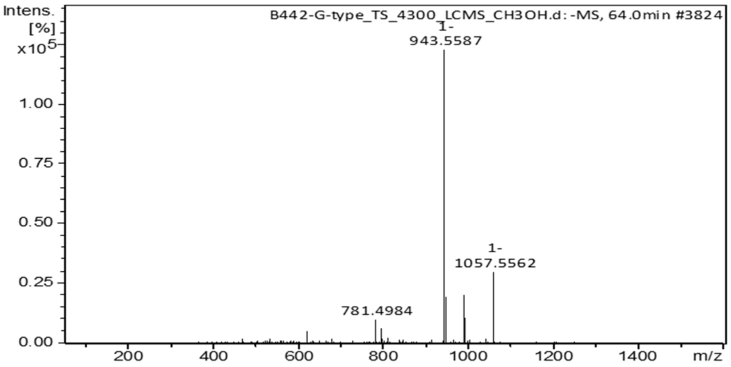

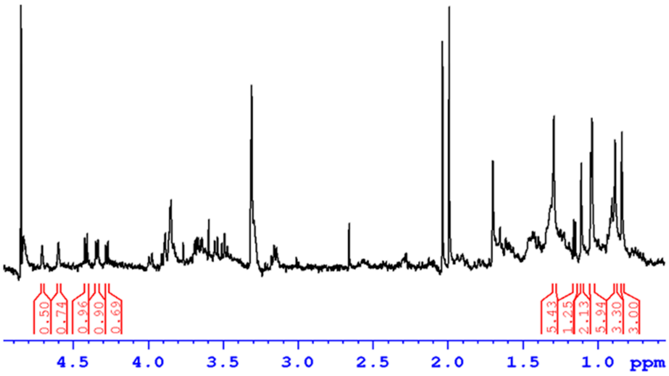

Figure S1. Mass spectral fragmentation pattern and 1D proton NMR spectrum of tentatively identified saponins measured in LC-SPE-NMR/MS experiment performed on saponin enriched extract of the G-type *B. vulgaris*. Tentatively identified P-type saponins correspond to peaks 17 (**A**), 18 and 20 (**B**), and 1 (**C**) in Table 2. Tentatively identified G-type saponins correspond to peaks 3 (**D**), 11 (**E**), and 12 (**F**) in Table 1.

U1: Quercetin-nTMS

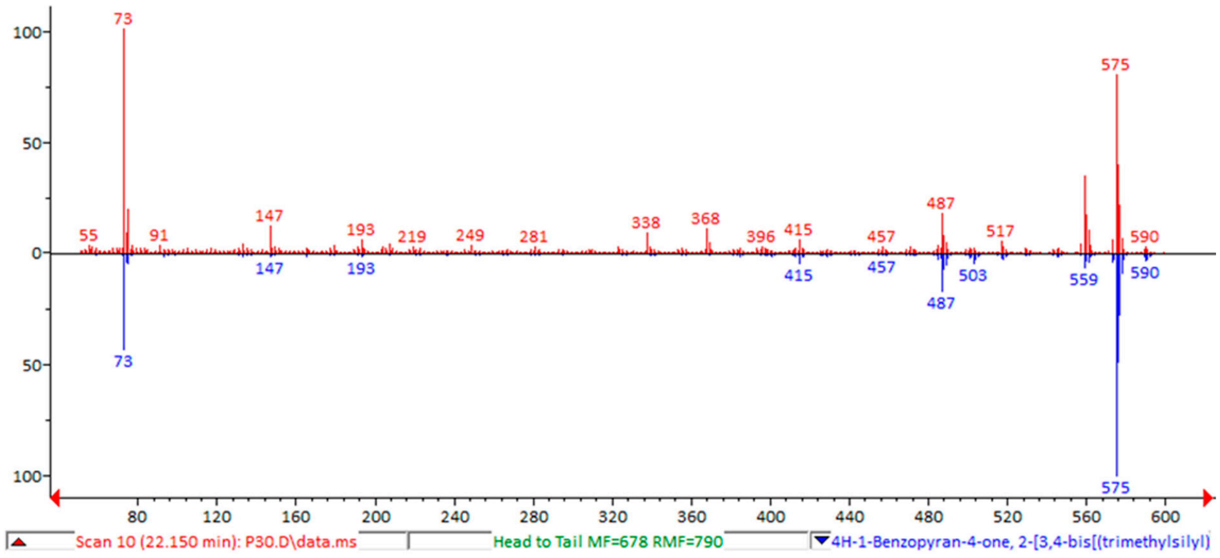

U2

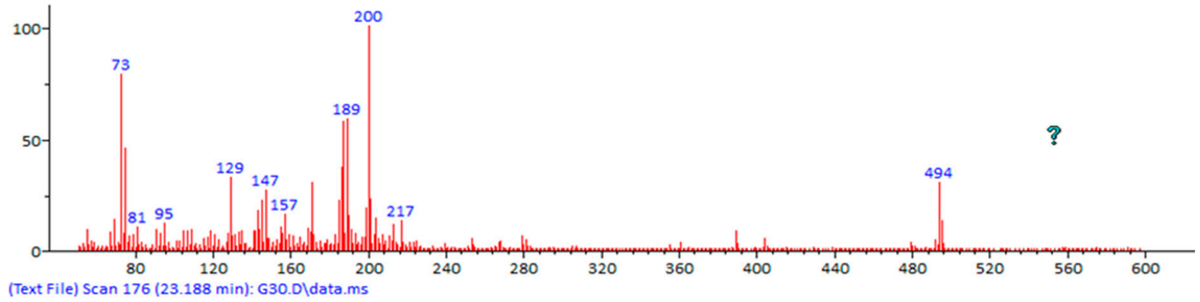

U3

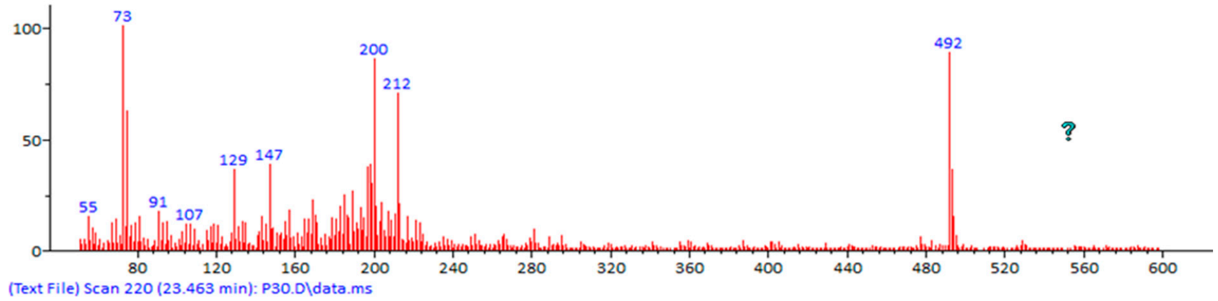

U4

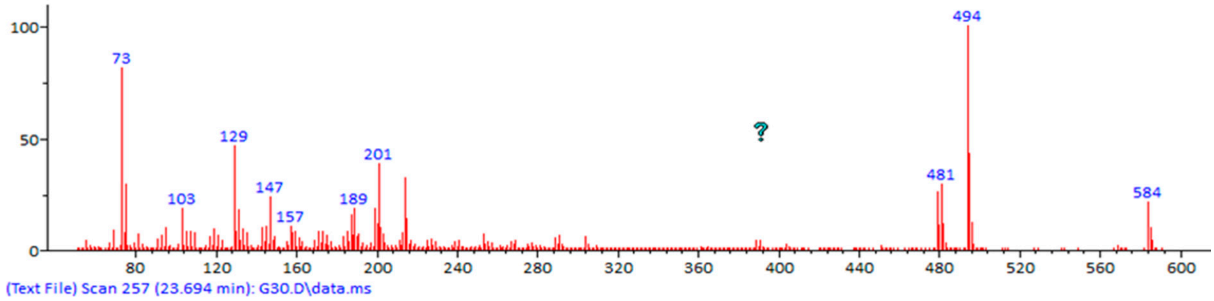
100
50
0
73
103
129
147
157
189
201
481
494
584
80
120
160
200
240
280
320
360
400
440
480
520
560
600
(Text File) Scan 257 (23.694 min): G30.D\data.ms

U5

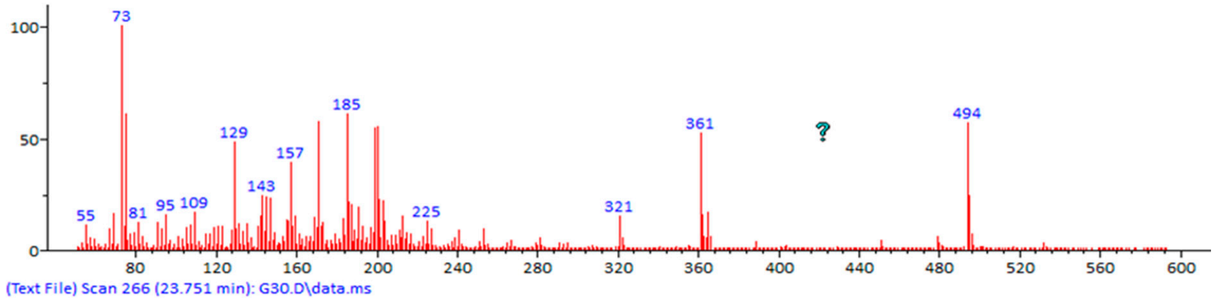
100
50
0
55
73
81
95
109
129
143
157
185
225
321
361
494
80
120
160
200
240
280
320
360
400
440
480
520
560
600
(Text File) Scan 266 (23.751 min): G30.D\data.ms

U6

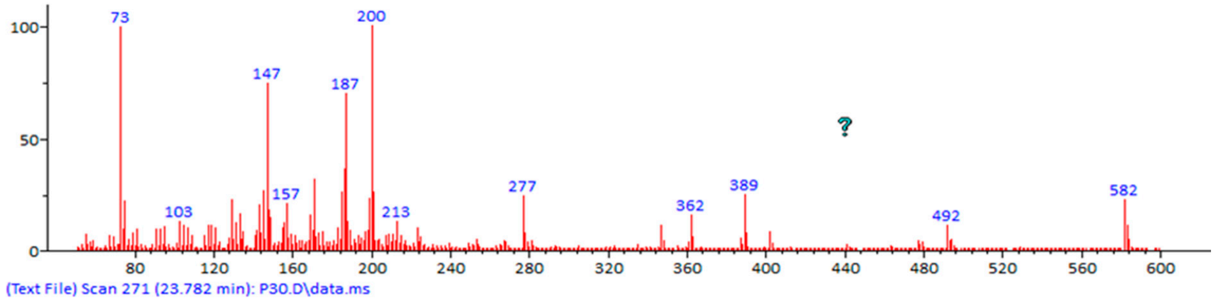
100
50
0
73
103
147
157
187
200
213
277
362
389
492
582
80
120
160
200
240
280
320
360
400
440
480
520
560
600
(Text File) Scan 271 (23.782 min): P30.D\data.ms

U7: Protopanaxatriol-nTMS

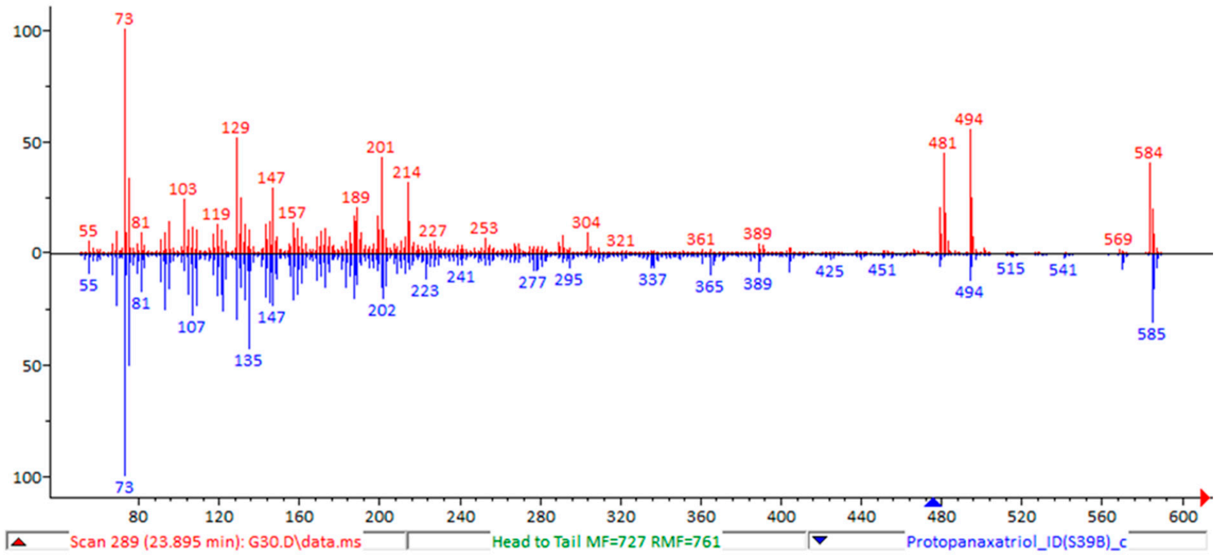

U8

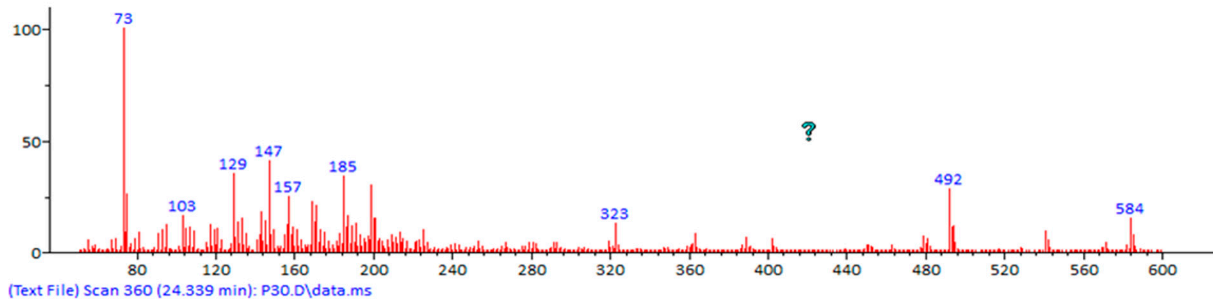

U9: Echinocystic acid-nTMS

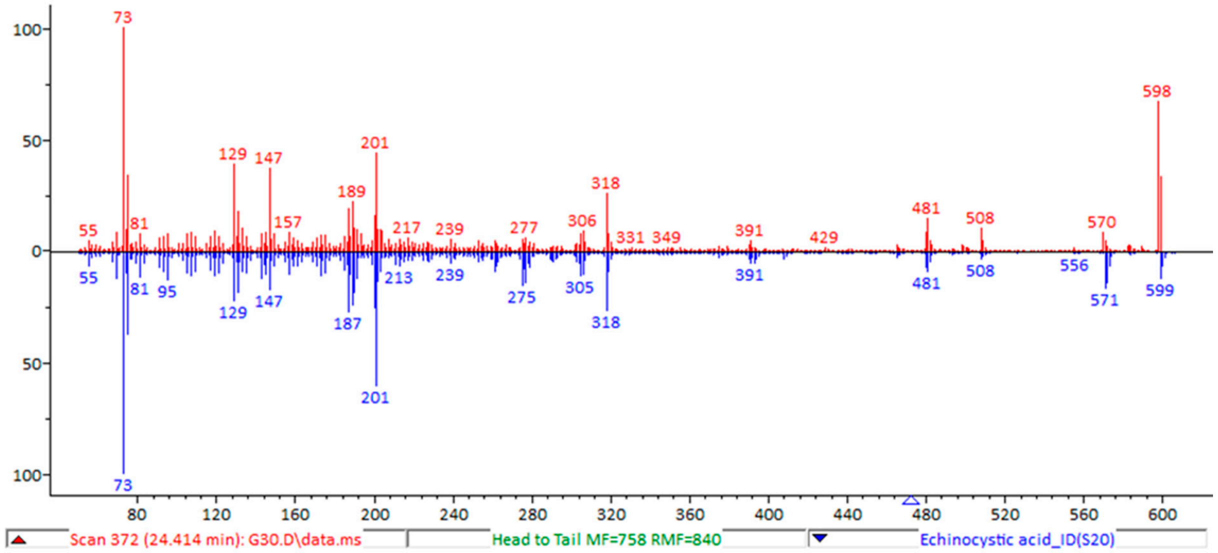

U10

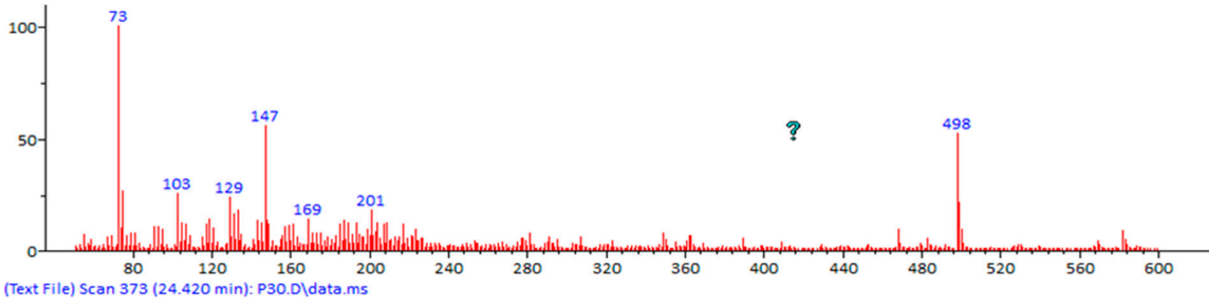

11: Oleanolic acid-2TMS

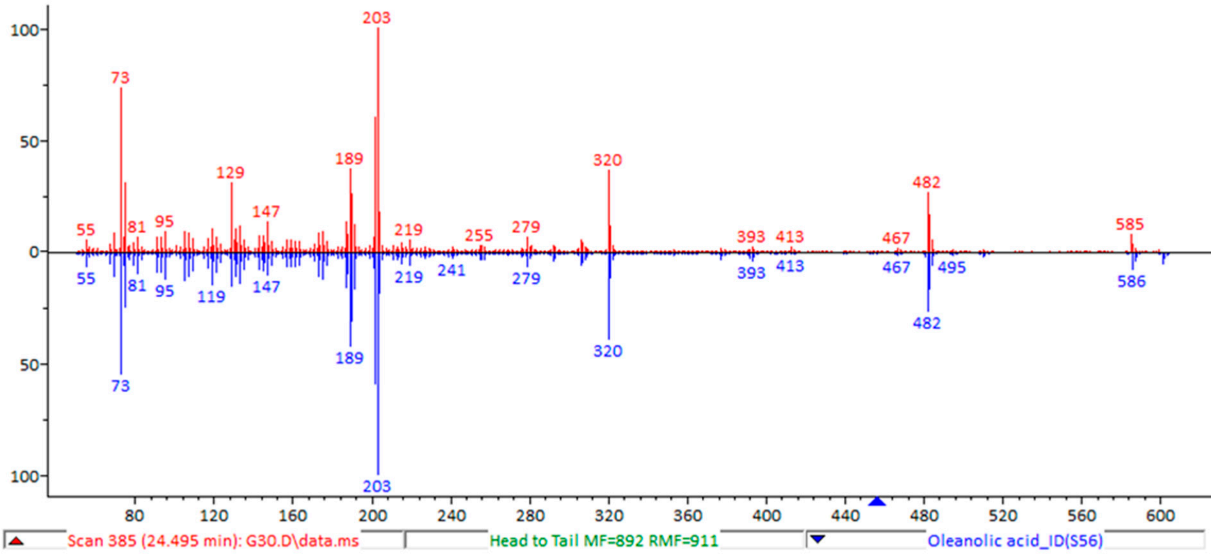

U12

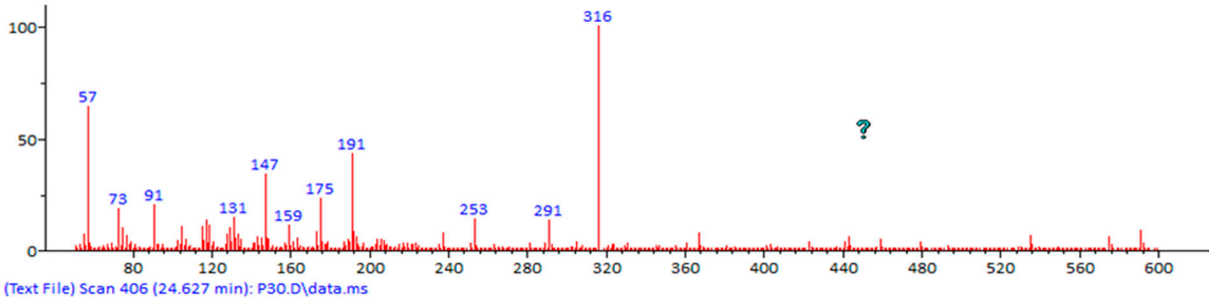

U13: Caulophyllogenin-nTMS

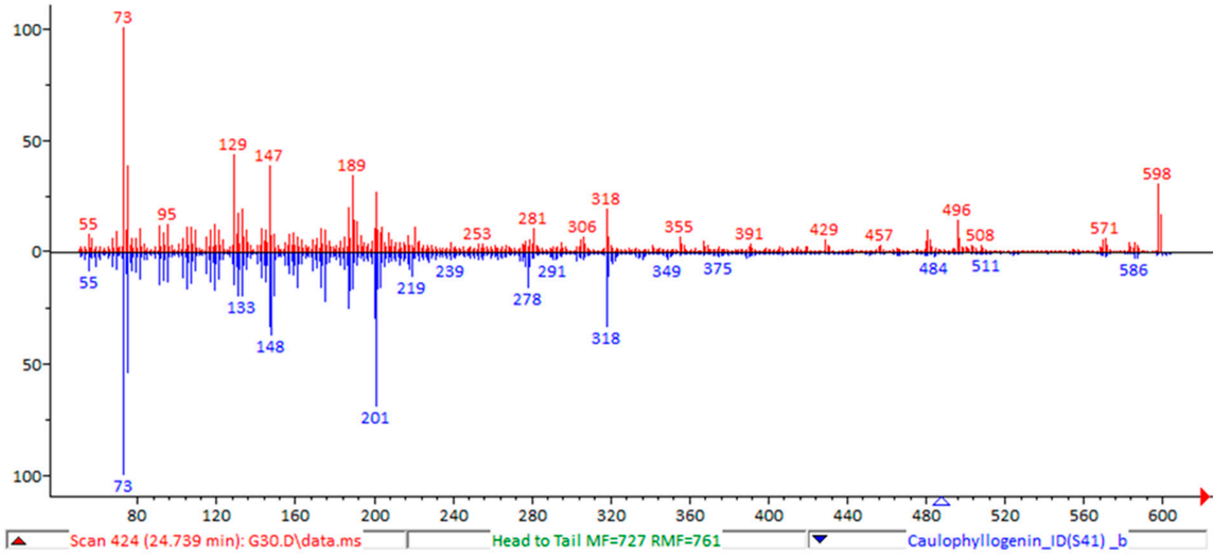

U14

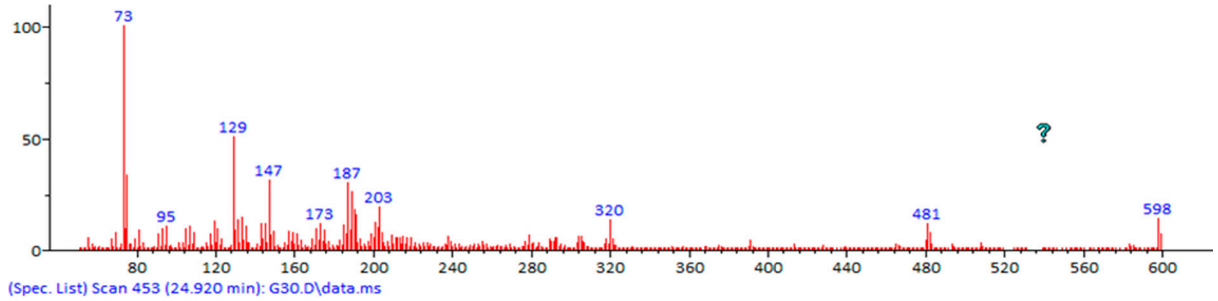

U15

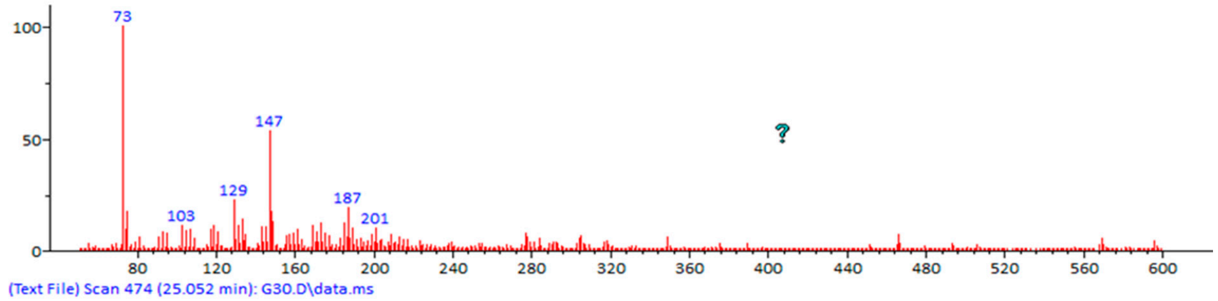

U16: Hederagenin-3TMS

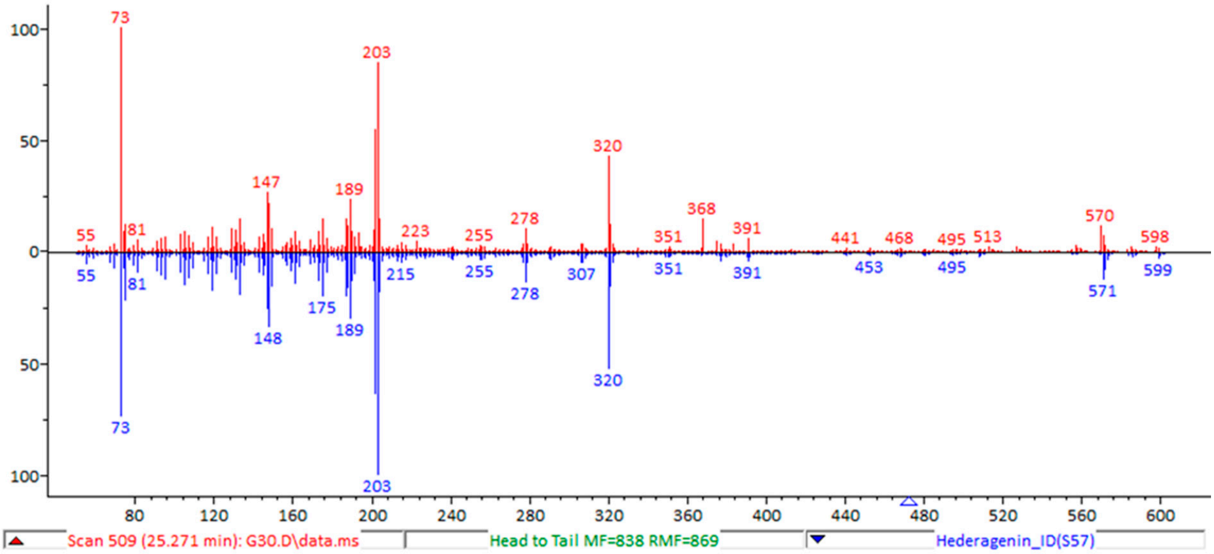

U17

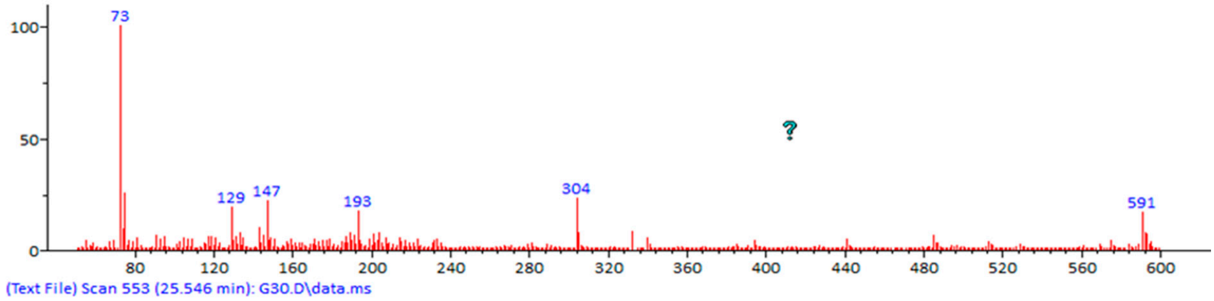

U18: Madecassic acid-nTMS

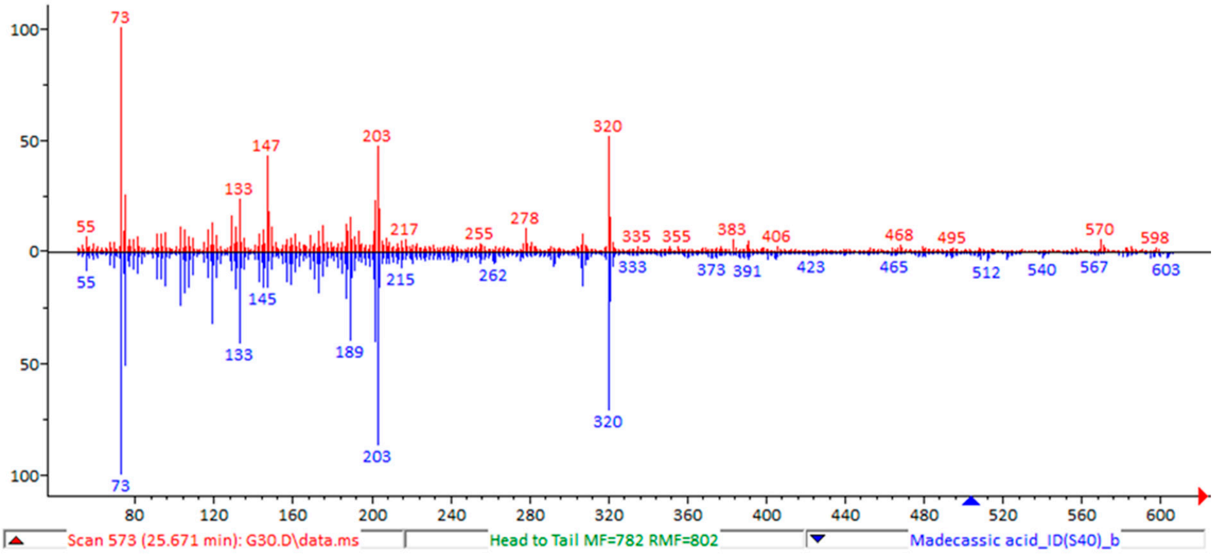

U19: Betulin-nTMS

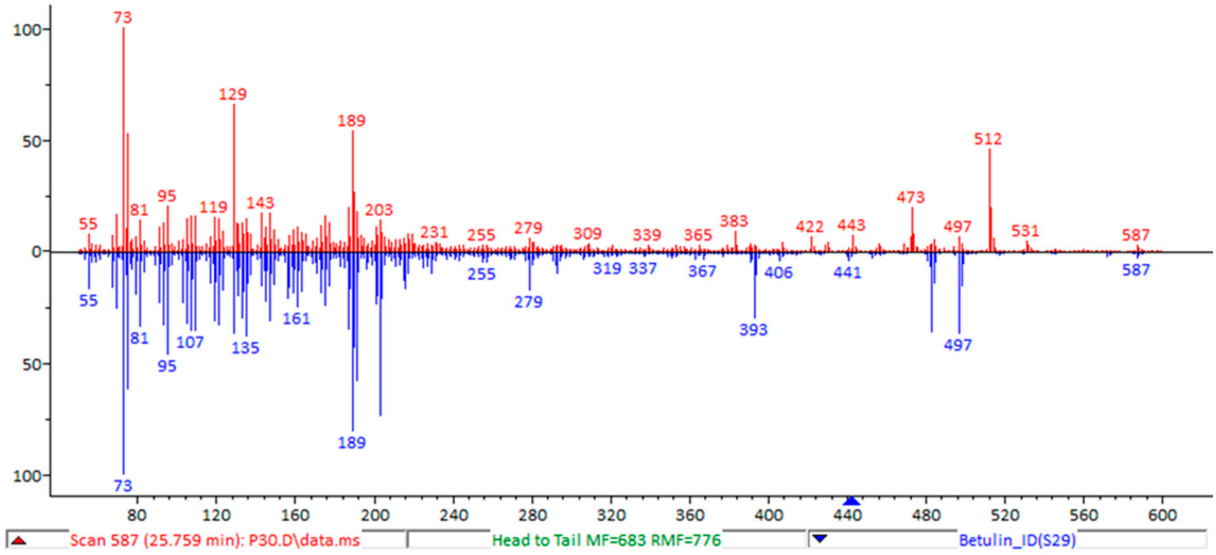

U20

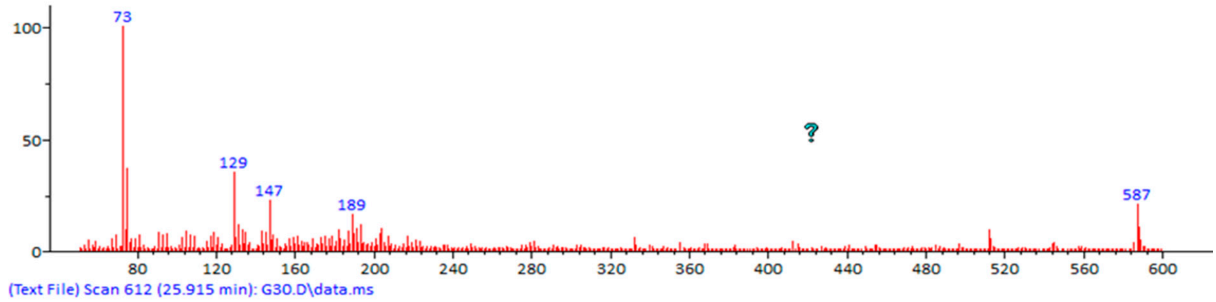

U21: Betulinic acid-nTMS

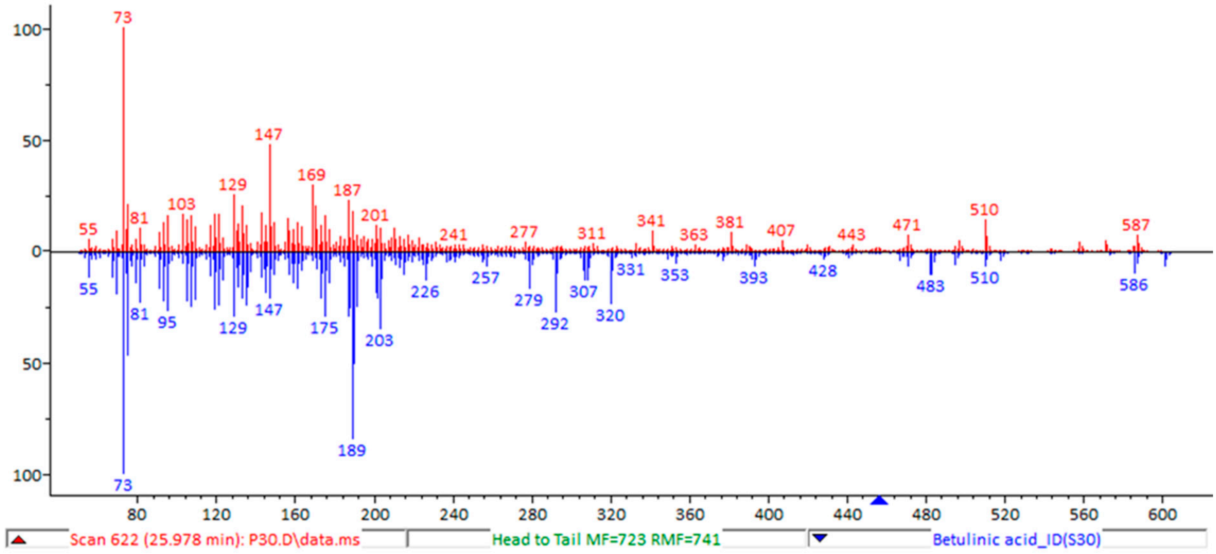

U22

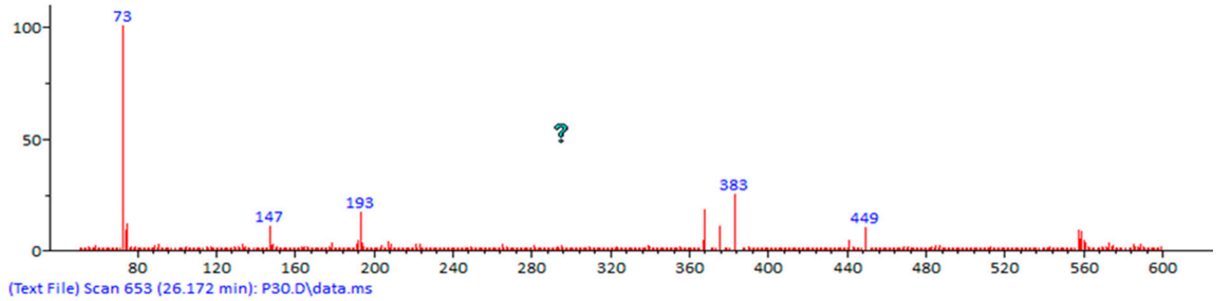

U23: 6-beta-maslinic acid-nTMS

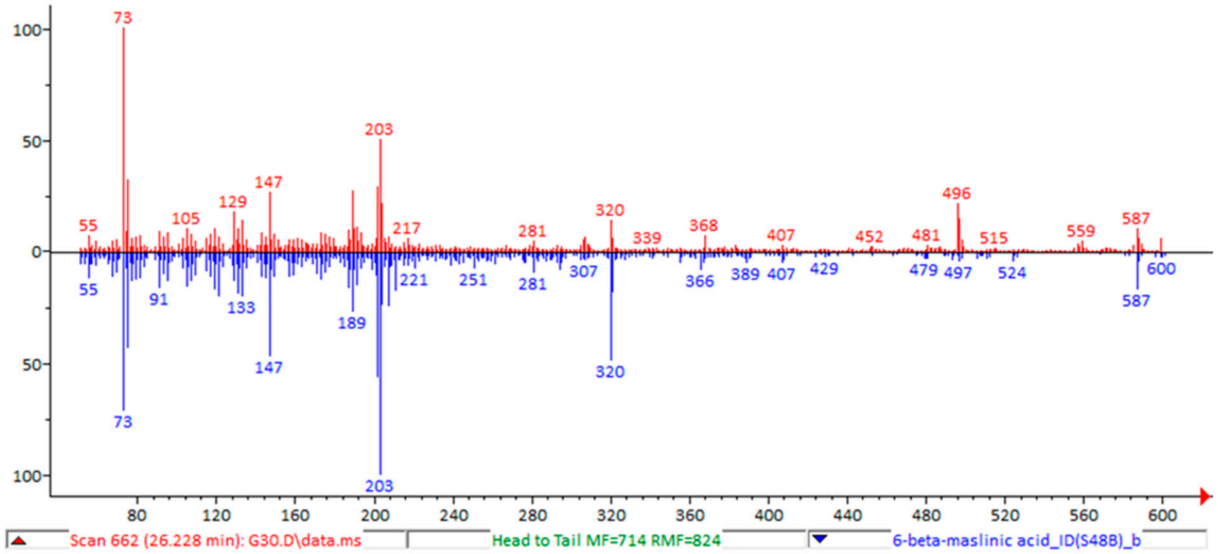

U24

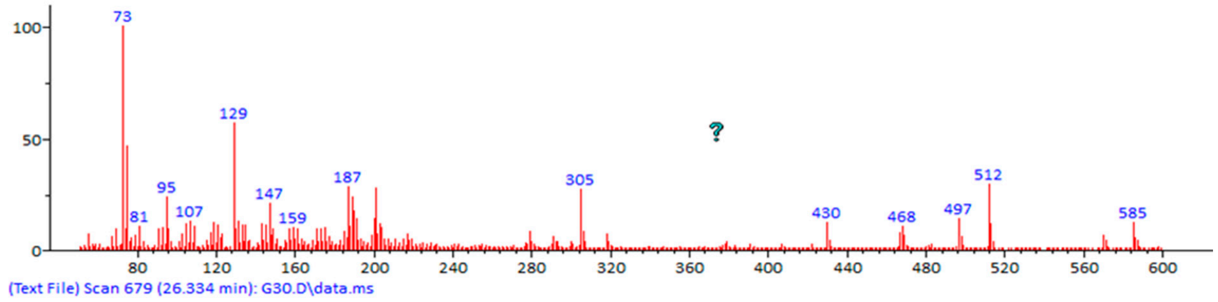

U25: Quillaic acid-nTMS

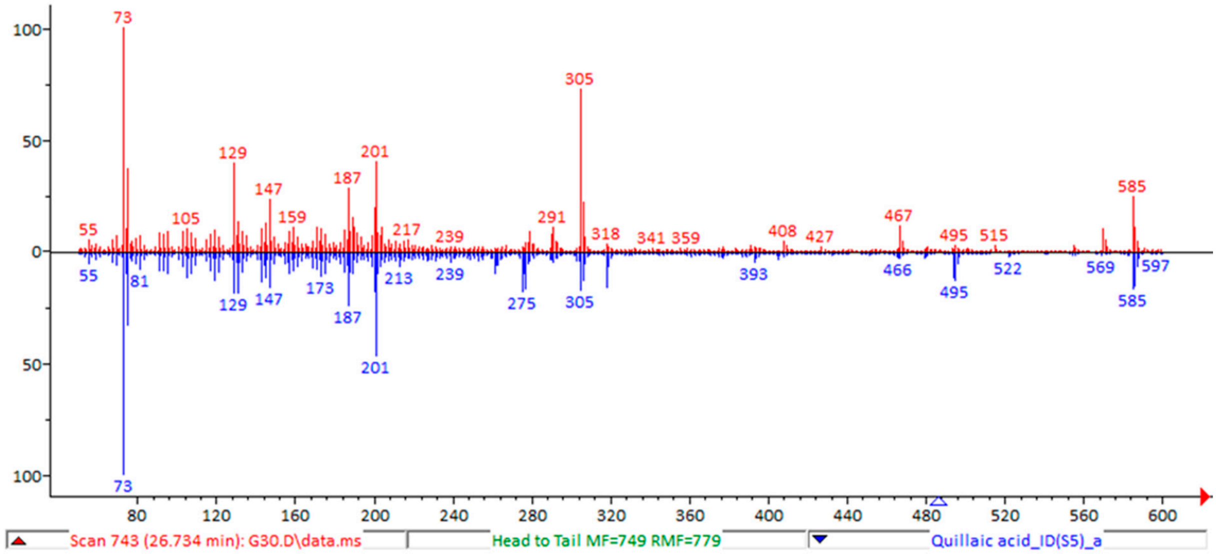

U26: Quillaic acid-nTMS

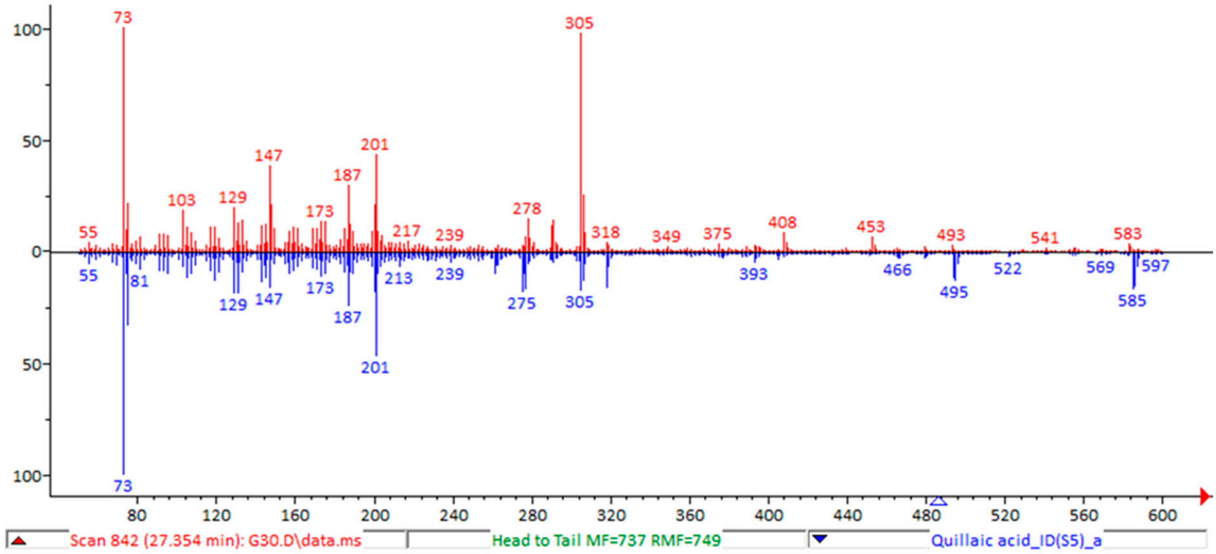

U27

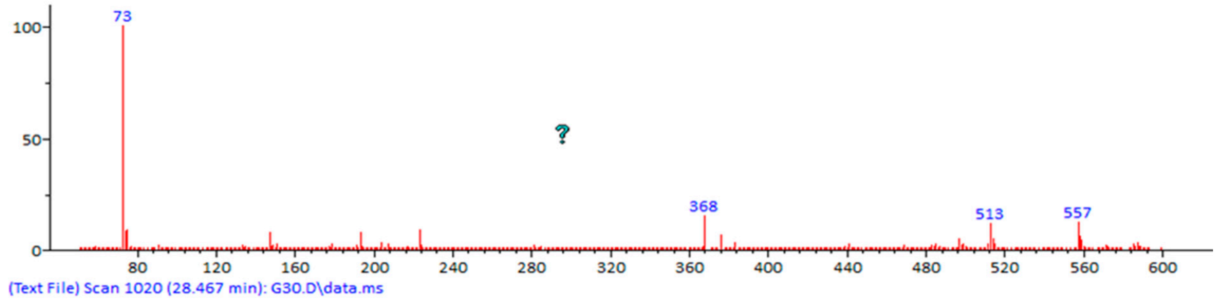

U28

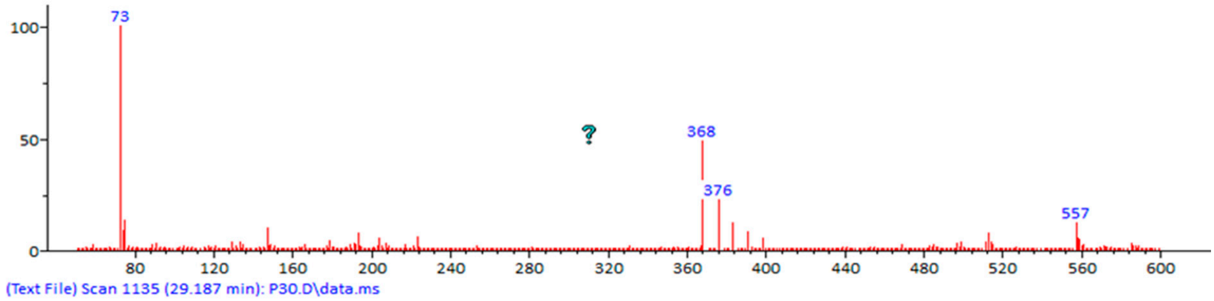

Figure S2. Electron impact-mass spectrum (EI-MS) fragmentation patterns of 28 peaks detected saponin enriched extracts of the G- and P-type *B. vulgaris* after acidic hydrolysis of the extracts which allowed cleave off sugar moieties attached to aglycones. GC-MS total ion current (TIC) chromatograms are illustrated in Figure 7.
